# Control of Skeletal Muscle Atrophy Associated to Cancer or Corticosteroids by Ceramide Kinase

**DOI:** 10.3390/cancers13133285

**Published:** 2021-06-30

**Authors:** Federica Pierucci, Alessia Frati, Chiara Battistini, Fabio Penna, Paola Costelli, Elisabetta Meacci

**Affiliations:** 1 Department of Experimental and Clinical Biomedical Sciences “Mario Serio”—Unit of Biochemical Sciences and Molecular Biology, University of Florence, Viale GB Morgagni 50, 50134 Florence, Italy; Federica.pierucci@unifi.it (F.P.); alessia.frati@unifi.it (A.F.); chiara.battistini@unifi.it (C.B.); 2 Department of Clinical and Biological Sciences, University of Turin, 10125 Torino, Italy; fabio.penna@unito.it (F.P.); paola.costelli@unito.it (P.C.)

**Keywords:** skeletal muscle mass wasting, cachexia, glucocorticoids, ceramide, ceramide kinase, sphingolipids, C2C12 skeletal muscle cells, atrogin-1/MAFbx

## Abstract

**Simple Summary:**

Skeletal muscle (SkM) represents the largest “organ” in the human body as it constitutes at least 40% of the body mass. SkM mass wasting is a serious conditions since pathological tissue degeneration significantly worsens the prognosis of the associated disease and significantly reduces the quality and life expectancy with an incidence of 10–50% in patients suffering of cancer, chronic infection and inflammation. Therefore, it is urgent to investigate the molecular basis of SkM mass wasting and identify potential therapeutic targets. Apart from cytokines and chemokines, also sphingolipid mediators, particularly sphingosine-1-phosphate and ceramide 1-phosphate (C1P), contribute to cancer and inflammation, however, the contribution of  ceramide kinase (CerK), and its product ceramide 1-phosphate (C1P), to SkM mass wasting in these conditions is missing. The present study was developed using mice bearing the C26, or Lewis lung carcinoma (LLC) tumors, well characterized models of cancer-associated SkM atrophy, and in murine and human myotubes treated with conditioned media obtained from C26 or LLC-cells or with the corticosteroid dexamethasone. The results obtained reveal that CerK protein expression and the Ceramide/C1P axis are markedly impaired in all experimental models, demonstrating that the CerK/C1P axis plays a crucial role as molecular regulator of SkM mass associated to cancer or corticosteroids.

**Abstract:**

Apart from cytokines and chemokines, sphingolipid mediators, particularly sphingosine-1-phosphate (S1P) and ceramide 1-phosphate (C1P), contribute to cancer and inflammation. Cancer, as well as other inflammatory conditions, are associated with skeletal muscle (SkM) atrophy, which is characterized by the unbalance between protein synthesis and degradation. Although the signaling pathways involved in SkM mass wasting are multiple, the regulatory role of simple sphingolipids is limited. Here, we report the impairment of ceramide kinase (CerK), the enzyme responsible for the phosphorylation of ceramide to C1P, associated with the accomplishment of atrophic phenotype in various experimental models of SkM atrophy: in vivo animal model bearing the C26 adenocarcinoma or Lewis lung carcinoma tumors, in human and murine SkM cells treated with the conditioned medium obtained from cancer cells or with the glucocorticoid dexamethasone. Notably, we demonstrate in all the three experimental approaches a drastic decrease of CerK expression. Gene silencing of CerK promotes the up-regulation of atrogin-1/MAFbx expression, which was also observed after cell treatment with C8-ceramide, a biologically active ceramide analogue. Conversely, C1P treatment significantly reduced the corticosteroid’s effects. Altogether, these findings provide evidence that CerK, acting as a molecular modulator, may be a new possible target for SkM mass regulation associated with cancer or corticosteroids.

## 1. Introduction

In adults, skeletal muscle (SkM) mass adjusts to diverse pathophysiological conditions by controlling pathways that regulate protein turnover [1,2,3]. In particular, endocrine and inflammatory factors, such as increased circulating glucocorticoids (GC), markedly play a part in SkM mass wasting and the pathogenesis of cancer-associated disorders, chronic inflammation or sepsis [4]. In this regard, a conspicuous amount of evidence supports the contribution of pro-inflammatory cytokines to cachexia, causing depletion of both adipose tissue and SkM mass, in view of their ability to inhibit anabolic pathways in favor of catabolic ones [5]. Along this line, experimental cachexia can be improved by specific anti-cytokine treatment strategies [4]. Moreover, since SkM tissue is the largest protein reservoir in the body, SkM loss is, in general, a poor prognostic indicator, impairing the efficacy of different therapeutic treatments and aggravating diseases leading to an increase of morbidity and mortality [1]. Therefore, it is urgent to investigate the molecular basis of SkM mass wasting and to identify potential therapeutic targets [4]. The reduction of SkM mass due to the presence of cancer or to GC treatment often results from increased protein degradation, mainly exerted through the hyperactivation of the autophagy-lysosome and the ubiquitin–proteasome proteolytic systems. The latter, in particular, relies on the specific up-regulation of transcripts encoding ubiquitin, ubiquitin-conjugating enzymes (E2), a few ubiquitin-protein ligases (E3) and several proteasome subunits [6,7,8,9]. Among the E3 family, only a few members are muscle specific and up-regulated during muscle protein depletion. In particular, the expression of well characterized muscle-specific ubiquitin ligases, namely atrogin-1/MAFbx, MuRF1/TRIM63 and, more recently, MUSA 1 and SMART [7,9,10,11,12] increases rapidly upon a variety of stressors, such as GC and pro-inflammatory cytokines [4,10], cancer cachexia and other pathological states [4,5].

Sphingolipids (SLs), first described as inactive and permanent structural elements of the cell membrane, are emerging as biologically active factors in different cell types, including SkM cells [13,14,15,16,17,18]. Several interconvertible SLs, such as ceramide (Cer), sphingosine (Sph), ceramide 1-phosphate (C1P) and sphingosine-1-phosphate (S1P), are able to control distinct cellular processes and functions, including carcinogenesis and inflammation [19,20,21,22]. In particular, C1P and ceramide kinase (CerK), play key roles in tumor promotion and dissemination. Interestingly, C1P can act as a pro-inflammatory as well as anti-inflammatory factor, depending on the biological systems [23].

Cer is formed via de novo synthesis or through sphingomyelin degradation, this latter catalyzed by sphingomyelinase. In mammals, Cer is used to produce C1P [24]. The inhibition of the de novo synthesis of Cer potentiates myoblast differentiation [25] and its accumulation promoted by Tumor Necrosis Factor-α (TNF-α) participates to myotube atrophy [26]. On the other hand, C1P has been reported to stimulate proliferation of different cell types [26,27,28] and to potently regulate cell migration and inflammatory responses [27,28,29]. Moreover, increased Cer levels have been reported in the SkM of tumor-bearing mice [26]. However, despite these observations, the contribution of both CerK and the Cer/C1P axis to muscle wasting associated to cancer and to corticosteroid response is not sufficiently explored.

Therefore, in order to evaluate the correlation between the CerK/C1P axis and the cachectic/atrophic phenotype, the present study was developed using: (a) mice bearing the C26 adenocarcinoma (C26) or the Lewis lung carcinoma (LLC), well characterized models of cancer cachexia [30,31,32]; (b) myotube cultures treated with conditioned media (CM) obtained from C26- or LLC-cells or with GC [32,33,34,35]. The results obtained reveal that CerK protein expression is markedly reduced in all experimental models of cancer-induced atrophy, demonstrating that the CerK/C1P axis plays a role of molecular regulator of SkM mass in these conditions.

## 2. Materials and Methods

### 2.1. Materials Biochemicals, Cell Culture Reagents

Dulbecco’s Modified Eagle’s Medium (DMEM), fetal calf serum (FCS), horse serum (HS), penicillin/streptomycin, protease inhibitor cocktail, bovine serum albumin, D-*erythro*-C8-ceramide (C8-cer, 10 μM) and NVP-231 (100 nM) competitive inhibitor of ceramide kinase (CerK) were purchased from Sigma Aldrich (Milan, Italy); C2C12 myogenic cells, human primary skeletal muscle cells, C26 adenocarcinoma and Lewis lung carcinoma cells were obtained from American Type Culture Collection (ATCC, Manassa, VA, USA); the bioactive sphingolipid ceramide 1-phosphate (C1P, 2 μM), dexamethasone (dexa, 100 μM), *N*-[(Phenylmethoxy)carbonyl]-L-leucyl-*N*-[(1*S*)-1-formyl-3-methylbutyl]-L-leucinamide MG132 (10 μM), proteasome and calpain inhibitor, K1 (iCK, 10 μM) CerK non-competitive inhibitor and N-[(1R,2S)-2-hydroxy-1-hydroxymethyl-2-(2-tridecyl-1-cyclopropenyl) ethyl]octanamide (GT11, 1 μM) dihydroceramide desaturase inhibitor were from Tocris (Bristol, UK); TRIzol reagent, Lipofecatmine 2000 Reagent, specific short hairpin RNAs (shRNAs) cloned into plKO.1-puro expression vector (Mission RNAi) were from Thermo Fisher Scientific (Carlsbad, CA, USA). Silencer Select Pre-designed CerK siRNA Product, high capacity cDNA-Reverse-Transcription kit and Syber Green reagent were from Life Technologies (Thermo Fisher Scientific, Carlsbad, CA, USA). protein concentration was determined by Bradford microassay (Bio-Rad, Hercules, CA, USA). Dry milk was from Bio-Rad, (Hercules, CA, USA). Amersham Hybond p 0,45 PVDF membrane and chemiluminiscence kit were from from GE Healthcare (Buckinghamshire, UK); goat polyclonal anti-atrogin-1/MAFbx was from ECM Biosciences (Versailles, KY, USA), rabbit polyclonal anti-CerK from abcam (Cambridge, UK). Goat polyclonal anti-SphK1 was from Sigma Aldrich (Milan, Italy), mouse monoclonal or rabbit polyclonal anti-β-actin and secondary antibodies coupled to horse radish peroxidase were from Santa Cruz Biotechnology (Santa Cruz, CA, USA). Goat polyclonal antibody to FBX-032 atrogin-1/MAFbx was from ThermoFisher Scientific (Invitrogen, Waltham, MA, USA) and Tali Cell Cycle Kit was from Life Technologies (Eugene, OR, USA).

### 2.2. Animals and Treatments

Balb/C mice (Charles River, Calco, Italy) ~20 g were kept on a dark-light cycle (light 8:00 to 20:00), with food and water during the duration of the experimental period. Randomized mice were divided into groups (controls and tumor bearers). The latter were inoculated subcutaneously in the back with 5 × 10^5^ C26 or LLC cells. Both cell lines were incubated in DMEM-10% FBS supplemented with antibiotics (penicillin, streptomycin), sodium pyruvate, L-glutamine (Sigma Aldrich, Milan, Italy), at 37 °C as reported in [17]. Tumor cells were suspended in PBS and implanted in the back of the animals.

Animal weight and food intake were registered daily. Control and tumor-bearing mice were sacrificed under anesthesia 14 days after C26 carcinoma or LLC implantation. Some tissues, among which the tibialis anterior muscle, were rapidly excised, weighed, frozen in liquid nitrogen and stored at –80 °C for additional analysis [36,37].

### 2.3. Plasmids and Tissue Electroporation

CerK silencing was achieved by using sequence-specific short hairpin RNAs (shRNAs). A set of shRNAs targeting CerK mRNA (CerK-shRNA) and cloned into plKO.1-puro expression vector was used (Mission RNAi, Thermo Fisher Scientific, Carlsbad, CA, USA). Three distinct plasmids contain the following oligonucleotide sequences (5′-3′): CATCGGCTTTGCACATCATTA; TCCAGTGGCCGATGGCATAAA; CGTTGAAGTTTAT CGAGTCAA. Plasmids were purified with a NucleoBond Xtra Maxi kit (Macherey-Nagel GmbH, Duren, Germany). The right tibialis anterior muscle was injected with 50 μg of plasmidic DNA, whereas the contralateral muscle served as control (SCR-shRNA). One minute after DNA injection, transcutaneous pulses were applied by two stainless steel plate electrodes. The electroporation was performed 10 days before animal sacrifice. With the transfection procedure described, no sign of muscle damage and inflammatory infiltrate could be seen on histological analysis [30].

### 2.4. Cell Culture and Treatments

Murine C2C12 skeletal myoblasts and human primary skeletal muscle cells obtained from ATCC (Manassas, VA, USA) were routinely grown in DMEM (Sigma Aldrich, Milan, Italy) with L-glutamine, penicillin/streptomycin (Sigma Aldrich, Milan, Italy), and 10% fetal bovine serum (FBS) (Sigma Aldrich, Milan, Italy), as suggested by the company. For C2C12 myoblasts cytometry analysis of the cell cycle distribution, cells were incubated for 24h with NVP-231 (100 nM Sigma Aldrich, Milan, Italy) competitive and K1 (iCK) (10 μM) (Tocris Bristol, UK) noncompetitive inhibitor of ceramide kinase (CerK), respectively [38,39].

For myotube experiments, myoblasts were induced to differentiation into myotubes as previously reported [17]. Briefly, C2C12 cells were seeded in 12- or 6-well plates (0.5 or 0.2 × 10^6^ cells/mL). At 90–100% confluence (72 h after seeding), the DMEM was replaced with differentiation medium (DM: DMEM containing 0.5% horse serum (HS) (Sigma Aldrich, Milan, Italy). The medium was changed every 48 h. Terminal differentiation was observed at 3–5 days after shifting the medium to DM. C2C12 myotubes were treated for 24 h with the inhibitor of dihydroceramide desaturase GT11, (10 μM) (Tocris, Avonmouth, Bristol, UK) [40]. Moreover, C2C12 myotubes were grown for 48 h with: MG132 (30 μM), proteasome and calpain inhibitor (Tocris, Avonmouth, Bristol, UK) C8-cer (10 μM) (Sigma Aldrich, Milan, Italy) [41] and with bioactive sphingolipid ceramide-1-phosphate (C1P) (2 μM) (Tocris, Avonmouth, Bristol, UK). After a 1 h incubation, myotubes were treated with the glucocorticoid dexamethasone (dexa, 100 μM) (Tocris, Avonmouth, Bristol, UK) for 48 h.

### 2.5. Conditioned Medium (CM) Collection

To collect conditioned media (CM), C26 and Lewis lung carcinoma cells were cultured in high-glucose DMEM (Sigma Aldrich, Milan, Italy), supplemented with 1% L-glutamine, 1% penicillin/streptomycin (Sigma Aldrich, Milan, Italy) and 10% fetal bovine FBS) (growth medium) (Sigma Aldrich, Milan, Italy), and maintained in a humidified atmosphere containing 5% CO_2_ at 37 °C [36,37]. Once the plates reached a confluency of >90%, after 24 h, the growth medium was collected and centrifuged at 4500 rpm for 20 min in 4 °C. Aliquots of the cell-cleared medium were stored at −80 °C. LLC and C26 conditioned media (CM-LLC; CM-C26) were diluted to 50–25–15% total volume in serum-free media. For the control group, 15–25% total volume of 10% fetal bovine serum growth media was diluted in serum free media as described in Brown et al., 2018 [35]. In all cases, serum content was standardized so that tumor and control cells had the same final serum concentration. C2C12 myotubes, plated on a 6-well plate, were then treated with either control or CM-LLC or CM-C26. At each 24 h interval after that, the medium was replaced with fresh control or new tumor cell-conditioned media for three days.

### 2.6. Cell Gene Silencing

CerK silencing was achieved by using the three specific shRNAs targeting CerK mRNA (CerK-shRNA), cloned into plKO.1-puro expression vector as described above. CerK silencing was also performed using sequence-specific short interfering RNA (siRNA) purchased from Life Technologies (Thermo Fisher Scientific, Carlsbad, CA, USA). The CerK RNA interference (CerK-siRNA) sequence starts at 142 nucleotides from the start codon (5′AGUUUAUCGAGUCAAGAAAtt3′ and 5′UUUCUUGACUCGAUA AACUtc3′) [42]. The specificity of CerK-siRNA was verified by sequence comparison with the human, mouse and rat genome database using the NIH BLAST program. C2C12 myotubes grown into 35-mm dishes (0.2 × 10^6^ cells/well) were transfected with CerK-siRNA (100 or 200 nM) using Lipofectamine 2000 reagent (1 mg/mL) according to manufacturer’s instructions (Thermo Fisher Scientific, Carlsbad, CA, USA). In preliminary studies, cell transfection with 200 nM CerK-siRNA was assessed to be maximally efficacious to down-regulate CerK. After 30 h from the beginning of transfection, cells were used for the experiments as previously described in [13,43].

### 2.7. Preparation of RNA and Measurement of Gene Expression by Quantitative Real-Time Polymerase Chain Reaction

CerK and atrogin-1/MAFbx mRNA levels were quantified by real-time PCR in murine C2C12 myotubes as well as in human myotubes. Specific primers for CerK, atrogin-1/MAFbx and the housekeeping Glyceraldehyde-3-Phosphate Dehydrogenase) gene (*GAPDH*) were designed using the program NCBI BLAST NUCLEOTIDE (Rockville Pike, Bethesda, MD, USA). *GAPDH* mRNA represents the endogenous control useful for the normalization of mRNA concentrations. Total RNA was prepared using TRIzol reagent (Thermo Fisher Scientific, Carlsbad, CA, USA). Quantity and quality of the RNA extracts were quantified using a Nanodrop (Thermo Scientific, Carlsbad, CA, USA). First-strand cDNA was synthesized in reverse transcription reaction (20 μL) with 0.5 μg RNA by using the high capacity cDNA-Reverse-Transcription kit (Life Technologies, Carlsbad, CA, USA). The reaction conditions were kept for 10 min at 25 °C, 120 min at 37 °C, 5 min at 85 °C. The Real-Time PCR reaction was performed as described in [17] using cDNA 100 ng, 0.25 μM of each primers (Primers stocks 100 μM, Sigma-Aldrich, Milan, Italy) and Power SYBR Green PCR Master Mix (Life Technologies, Carlsbad, CA, USA). The gene amplification was conducted in an MIC (Diatech Pharmacogenetics, Jesi, AN, Italy): 95 °C for 10 min, followed by 40 cycles at 95 °C for 30 s, 49–53 °C for 30 s, 72 °C for 45 s, 95 °C for 15 s, 60 °C for 60 s, 95 °C for 15 s and 60 °C for 15 s. The sequences of the primers are here listed: *GAPDH* forward: 5′-GGCAAATTCAACGGCACAGTC-3′, reverse 5′-TCGCTCCTGGAAGATGGTG-3′; atrogin-1/MAFbx forward: 5′-GCAGCCAAGAAGAGAAAGAA-3, reverse 5′-CTGTGACTTTGCTATCAGC-3′; CerK forward: 5′-GGACTTAATC AAGGACAGTGA-3′, reverse 5′-AGGACA GTGTCCCTTCATAG-3′. Both positive and negative controls were included in the analysis. Relative CerK and atrogin-1/MAFbx transcript levels were calculated: fold = 2^−∆∆Ct^ as in [17] (∆Ct, difference in Ct between the target gene and the housekeeping one; ∆∆Ct, difference between the ∆Ct of the interest sample and the ∆Ct of the reference one). mRNA levels in controls were arbitrarily set to 1.0.

### 2.8. Western Blot Analysis

Protein levels of atrogin-1/MAFbx, CerK was determined by Western blotting using myotubes and Balb/C mice tissue as in [17]. Briefly, cells were lysed with RIPA buffer (50 mM TrisHCl, pH 7.5, 120 mM NaCl, 1 mM EDTA, 6 mM EGTA, 15 mM Na_4_P_2_O_7_, 20 mM NaF, 1% Nonidet) and protease inhibitor cocktail (Sigma-Aldrich, Milan, Italy). Lysates were centrifuged at 600× *g* for 6 min at 4 °C, and protein concentration was measured using the Bradford microassay (Bio-Rad, Hercules, CA, USA). For cell membrane preparation, cell lysates were further centrifuged at 45,000× *g* for 1 h and pellet resuspended in RIPA buffer. Aliquots (40 μg) were diluted in 2X loading buffer (Sigma-Aldrich, Milan, Italy) and boiled at 90 °C for 10 min. Samples were subjected to 10% SDS-polyacrylamide gel electrophoresis and blotted on an Amersham Hybond PVDF membrane (GE Healthcare, Buckinghamshire, UK). The blots, washed with PBS, were blocked with PBST buffer containing 5% non-fat dry milk (Bio-Rad, Hercules, CA, USA). After blocking, the membranes were washed again and incubated overnight with primary antibody (1:1000 dilution), which contained goat polyclonal antibody to SphK1 (Sigma-Aldrich, Milan, Italy), goat polyclonal antibody to atrogin-1/MAFbx (ECM biosciences, Versailles, KY, USA) or goat polyclonal antibody to FBX-032 atrogin-1/MAFbx (ThermoFisher Scientific, Invitrogen, Waltham, MA, USA), rabbit polyclonal antibody to CerK (Abcam, Cambridge, UK) and mouse monoclonal antibody (Santa Cruz Biotechnology, Inc., Santa Cruz, CA, USA) or rabbit polyclonal (Sigma-Aldrich, Milan, Italy) to β-actin. After washing with PBST, the blots were incubated with goat anti-rabbit or goat anti-mouse or bovine anti-goat horseradish peroxidase-conjugated secondary antibody (Santa Cruz Biotechnology, Inc., Santa Cruz, CA, USA) at a 1:10,000/1:5000 dilution (1 h at room temperature) and developed as in [17], using ECL Western Blotting Detection Reagents (GE Healthcare, Buckinghamshire, UK), high performance chemiluminescence film (GE Healthcare, Buckinghamshire, UK) or Chemidoc (Amersham, Healthcare, Buckinghamshire, UK). The identity was confirmed by comparing to the molecular weight marker (BioRad, Hercules, CA, USA). Densitometric analysis of the bands was determined using ImageJ software (Bethesda, MD, USA) and Quantity-One (Imaging and Analysis Software by Bio-Rad Laboratories, Hercules, CA, USA) as in [17], and intensity was reported as relative percentage (means ± SEM), calculating the ratio of specific protein on β-actin and normalizing to control, set as 1 [13,44,45]. Full uncropped Western blot figures can be found at Figures S1–S13.

### 2.9. Cell Cycle Analysis

Cell cycle analysis was performed as in Pierucci et al. [45]. Briefly, C2C12 myoblasts were collected, washed once with DPBS (Sigma Aldrich, Milan, Italy) and fixed with ice-cold 70% ethanol in dH_2_O (Sigma Aldrich, Milan, Italy) to a concentration of 1 × 10^6^ to −5 × 10^6^ cells/mL. The samples were placed at least for a night at –20 °C, then ethanol was removed, and cells were washed with DPBS + 1%BSA (Bio-Rad, Hercules, CA, USA). After DPBS was removed, cells were stained with propidium iodide incubating them at room temperature for 30 min in the dark with the Tali^®®^ Cell Cycle Solution (Life Technologies, Eugene, OR, USA) to a concentration of 1 × 10^5^ to –5 × 10^6^ cells/mL. Cell suspension (25 µL) was transferred in the dedicated slide and cell cycle analysis was performed using the Tali^®®^ Image-Based Cytometer (Thermo Fisher Scientific, Carlsbad, CA, USA). The percentage of cells in each phase of the cell cycle was determined using FCS Express Research Edition software (version 4.03; De Novo Software, Morristown, NJ, USA).

### 2.10. Presentation of Data and Statistical Analysis

Results are expressed as mean ± SEM of at least 3 independent experiments. Statistical significance was determined by two-sided Student’s t-test, with a value of *p* < 0.05 considered significant. In multiple comparisons, statistical significance was determined using ANOVA and Newman–Keuls post-test, Pearson Index. Calculations were performed using GraphPad INSTAT + 3.3 software (GraphPad, San Diego, CA, USA).

### 2.11. Study Approval

Animals were cared for in compliance with the Policy on Human Care and Use of Laboratory Animals (ILAR 2011). The Bioethical Committees of the University of Turin approved the experimental protocol. All animal manipulations were made in accordance with the European Community guidelines for the use of laboratory animals (NIH, 1996). Ethical approval code: Italian Ministry of Health (Aut. Nr. 579/2018-PR).

## 3. Results

### 3.1. CerK Expression Is Reduced in the SkM of the C26 and LLC Hosts

**Figure 1:**
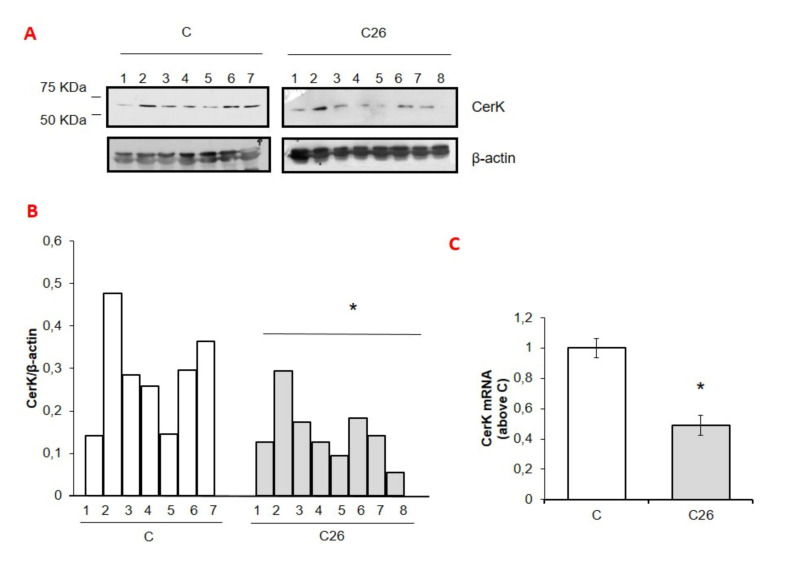

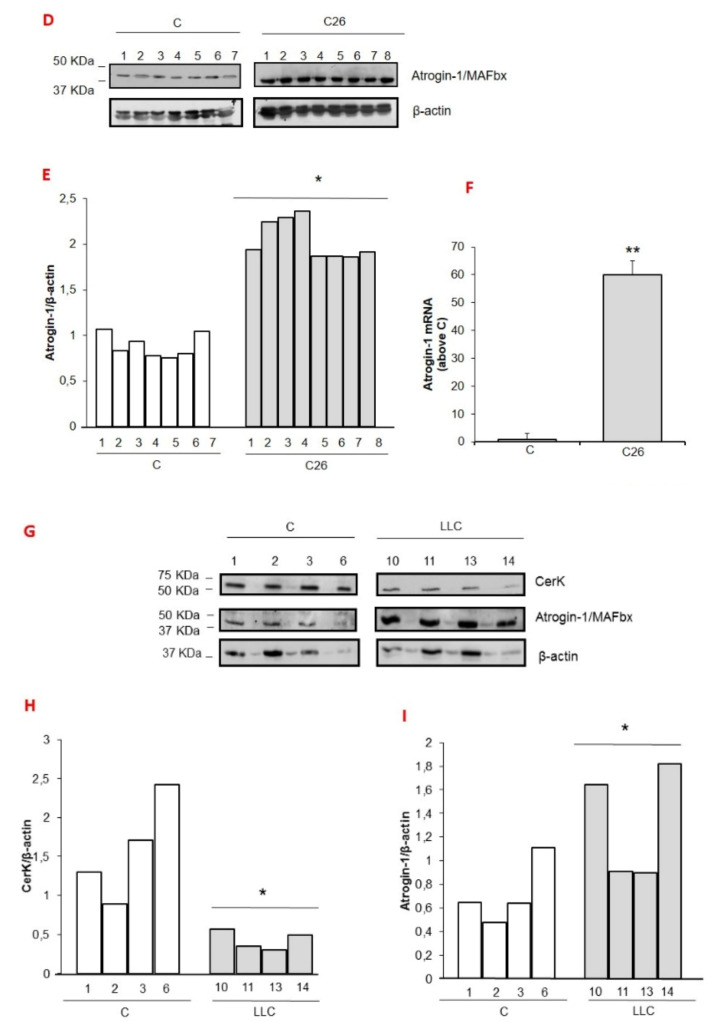
CerK or atrogin-1/MAFbx protein and mRNA expression in SkM tissues of cachectic C26 adenocarcinoma and Lewis lung cells bearing-mice (**A**–**I**). Cell lysates for CerK or atrogin-1/MAFbx protein expression and total RNA for CerK or atrogin-1/MAFbx mRNA expression were determined from tibialis muscle tissues of healthy and cachetic C26 adenocarcinoma (C26) and Lewis lung cells (LLC) bearing-mice as described in Methods. Mice were randomized into two groups, namely C (controls, *n* = 7) and C26 (*n* = 8) or C (controls, *n* = 4 and LLC hosts (*n* = 4). Aliquots (40 µg) of lysates obtained from tibialis muscle of healthy (**C**) and cachetic (C26/LLC) tumor-bearing mice were subjected to SDS-PAGE and immunoblotted with specific anti-CerK (**A**,**G**) or anti-atrogin-1/MAFbx (**D**,**G**) antibodies. A blot representative of at least three independent experiments and densitometric analysis of CerK (**B**,**H**) or atrogin-1/MAFbx (**E**,**I**) is shown. Data (means ± SEM), normalized to the β-actin band, are reported in the graph relative to specific controls (Student’s *t*-test, * *p* < 0.05 vs. C) (**C**,**D**,**F**). Total RNA was purified from tibialis muscle tissue of healthy and C26 cachetic mice and reverse transcripted as in Methods. Real-time PCR was performed using specific primers for CerK (**C**) or atrogin-1/MAFbx (**F**). Data, presented as fold change (mean ± SEM) of at least 3 independent experiments are shown (Student’s *t* test,* *p* < 0.05, ** *p* < 0.01 vs. C). Pearson index −0.08 in C and −0.53 in C26 tumor bearing mice).

Conforming the data reported in [30,31], the growth of the C26 tumor in mice is associated with reduced body weight and loss of both SkM and adipose tissue mass (Table 1). As shown in Figure 1, CerK expression in the gastrocnemius muscle of the C26 hosts was significantly lower than in controls (C), at both mRNA and protein levels (Figure 1A–C). Such reduction was paralleled by a marked increase of atrogin-1/MAFbx (Figure 1D–F), an accepted marker of protein degradation. These observations suggest that CerK and atrogin-1/MAFbx expression are inversely correlated (Pearson index −0.08 in C and −0.53 in C26 hosts).

Notably, similar results were obtained using SkM tissue of LLC-bearing mice, a different experimental model of cancer cachexia (Figure 1G–I) [32].

### 3.2. CerK Expression in C2C12 Cultures

**Figure 2:**
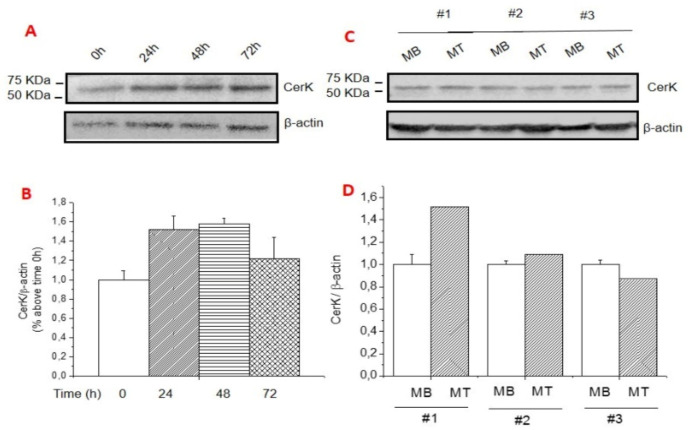

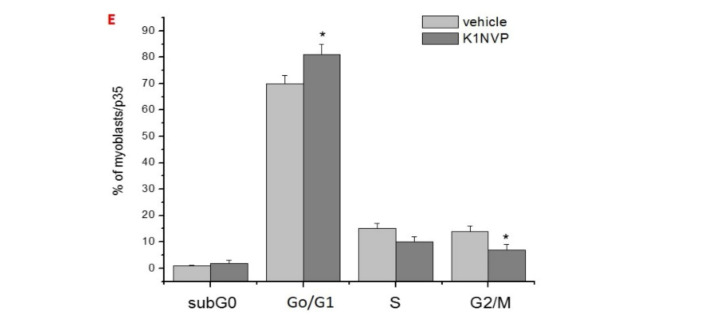
Expression CerK in differentiating C2C12 myoblasts and terminally differentiated myotubes, and effect of CerK inhibitors on cell cycle progression. (**A**–**D**): CerK expression in C2C12 cells at different stages of cell growth and myogenic differentiation process. Cell lysates were obtained from 100% confluent (t0) myoblasts (MB) and from cells incubated for the indicated time points (24 h, 48 h, 72 h) in differentiation medium containing 0.5% horse serum, and from differentiated myotubes (MT). Lysate proteins (30 µg) were separated by SDS-PAGE and immunoblotted with anti-CerK antibodies (**A**,**C**). A blot representing the densitometric analysis of CerK (**B**,**D**) is shown. Normalized data (mean ± SEM) to the â-actin band are reported in the graph relative to 100% confluent (t0) MB. (**E**): Cytometric analysis of the cell cycle distribution. C2C12 myotubes were treated with the noncompetitive CerK inhibitor, K1 (10 ìM) and the competitive inhibitor NVP-231 (100 nM) for 24 h. Cells were collected and counted by TALI^®®^ Cytometer as reported in Methods. Histograms show the percentage of cells in the specific subG0, G0/G1, S and G2/M phases. Three independent experiments in triplicate were performed. Data (means ± S.E.M.) reported in the graph are relative to untreated cells (vehicle). Student’s *t*-test, * *p* < 0.05.

To examine the molecular mechanisms able to modulate CerK expression in SkM wasting, C2C12 myotube cultures were exposed to dexa. This is one of the model systems more frequently used to mimic in vitro the phenotype occurring in pathological states characterized by systemically driven SkM wasting [33,34]. Figure 2A–D reports that in the absence of dexa treatment, the expression of CerK was maintained constant during the differentiation from myoblasts to myotubes (Figure 2A–D). The relevance of physiological CerK levels to myogenic homeostasis is supported by the observation that pharmacological inhibition of CerK promoted myoblast cell growth arrest (Figure 2E) and resulted in myotube reduction in size.

**Figure 3:**
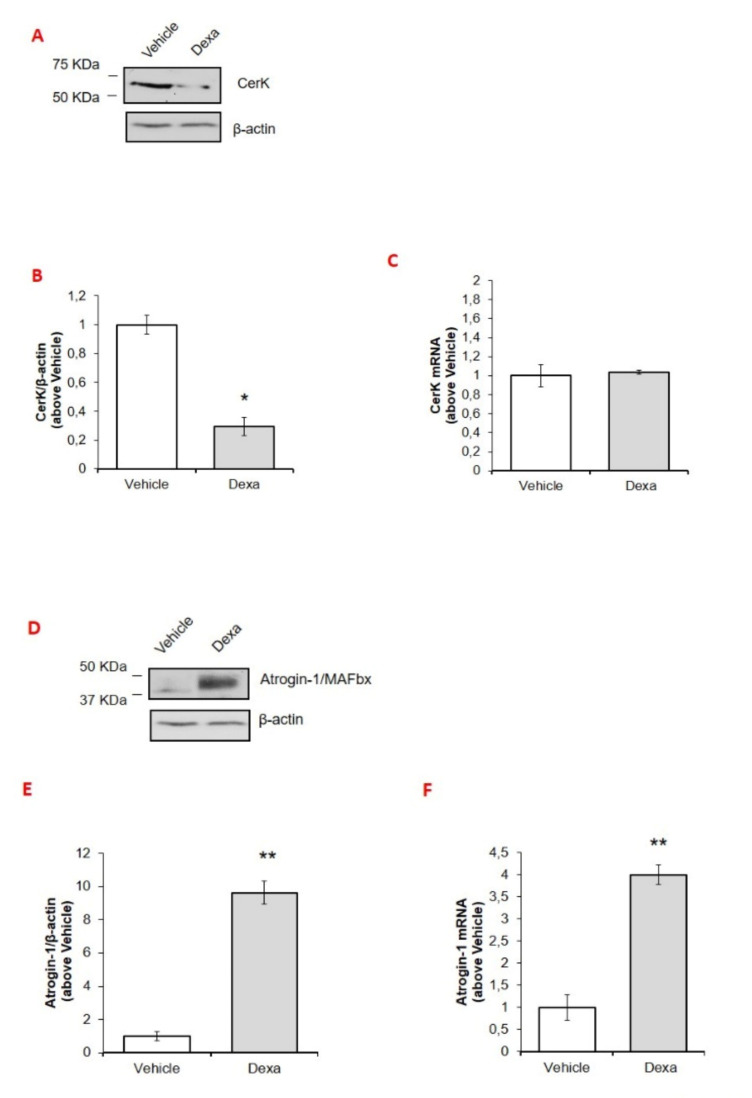

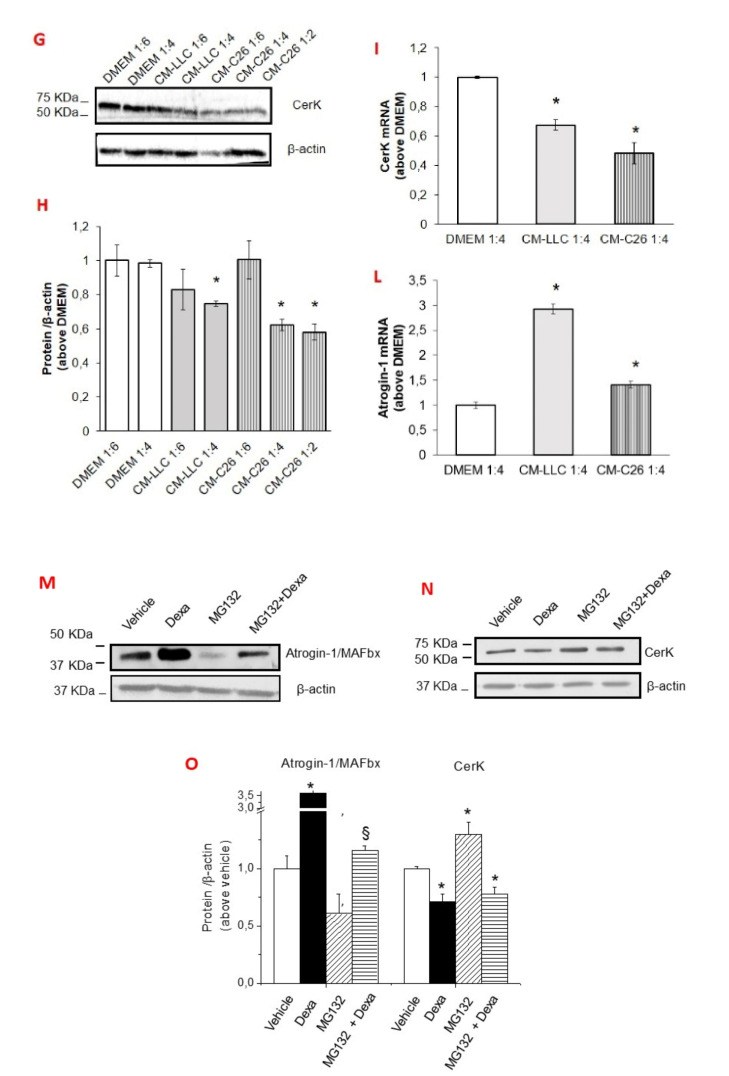
Effect of desamethasone or of conditioned medium derived from LLC or C26 cells of MG132 on CerK and atrogin-1/MAFbx protein and mRNA expression in C2C12 myotubes. (**A**–**F**): effect of desamethasone on CerK and atrogin-1/MAFbx expression in C2C12 myotubes. Cell lysates and total RNA for CerK and atrogin-1/MAFbx mRNA and protein expression quantification were obtained from untreated (vehicle, DMSO less than 0.05%) or dexamethasone-treated myotubes (Dexa) as described in Methods. C2C12 cell lysates (40 µg) obtained from vehicle- and dexa-treated (100 μM dexa) for 48 h were subjected to SDS-PAGE and immunoblotted with specific anti-CerK (**A**) or anti-atrogin-1/MAFbx (**D**) antibodies. A blot representative of at least three independent experiments and densitometric analysis of CerK (**B**) or anti-atrogin-1/MAFbx (**E**) in vehicle- or dexa-treated myotubes is shown. Data (means ± SEM) were normalized as reported in the legend of Figure 2 (Student’s *t* test, * *p* < 0.05, ** *p* < 0.01 vs. vehicle). (**C**,**F**): total RNA was purified and reverse transcripted as in Methods. Real-time PCR was performed using primers for CerK (**C**) or atrogin-1/MAFbx (**F**). Data are presented as fold change (mean ± SEM) of at least three independent experiments (Student’s *t* test, ** *p* < 0.01 vs. vehicle). (**G**–**I**): effect of conditioned medium derived from LLC cells or C26 cells on CerK and atrogin-1/MAFbx expression. Cell lysates for CerK protein expression and total RNA for CerK and atrogin-1/MAFbx mRNA quantification were obtained from myotubes cultured with DMEM (control group) or with conditioned medium obtained from LLC (CM-LLC) or C26 cells (CM-C26) as described in Methods. C2C12 cell lysates (40 µg) obtained from untreated and CM-treated myotubes for 72 h were subjected to SDS-PAGE, blotted and CerK immunodetected (**G**) with specific anti-CerK antibody. A blot representative of at least three independent experiments and densitometric analysis of CerK in DMEM or CM-treated myotubes is shown in (**H**). Data (means ± SEM) were normalized as reported as above (Student’s *t* test, * *p* < 0.05 vs. DMEM). (**I**,**L**): total RNA was purified and reverse transcripted as in Methods. Real-time PCR was performed using primers for CerK (**I**) and atrogin-1/MAFbx (**L**). Data are presented as fold change (mean ± SEM) (Student’s *t* test, * *p* < 0.05 vs. vehicle, ** *p* < 0.01 vs. DMEM). (**M**–**O**): effect of MG132, inhibitor of the proteasome and calpain on CerK expression. Myotubes were incubated with vehicle (DMSO less than 0.05%) or MG132 (30 μM) prior 100 μM dexamethasone (Dexa) for 48 h. Protein expression was detected in cell lysates (30 µg by SDS-PAGE and immunoblot using specific anti-atrogin-1/MAFbx (**M**) or anti-CerK (**N**) antibodies. A blot representative of three independent experiments and densitometric analysis of atrogin-1/MAFbx or CerK in vehicle- and dexa- or MG132-treated myotubes as reported above is shown (**O**). Data (mean ± SEM) normalized to the â-actin band are reported in the graph relative to vehicle (Student’s *t* test, * *p* < 0.05 vs. vehicle; § *p* < 0.05 vs. MG132).

When C2C12 myotubes were exposed to dexa, CerK protein levels were dramatically reduced (Figure 3A,B), whereas atrogin-1/MAFbx expression was increased (Figure 3D,E), thus recapitulating the pattern taking place in the SkM of both the C26 and the LLC hosts. By contrast, the same was not occurring at the mRNA level, where the increase of atrogin-1/MAFbx (Figure 3F) was not paralleled by modulations of CerK expression (Figure 3C), suggesting a post-translational regulation of CerK protein levels in C2C12 myotubes.

Reduced CerK protein and mRNA levels and increased atrogin-1/MAFbx expression also occurred when C2C12 myotubes were incubated with the conditioned medium (CM) obtained from LLC or C26 cells (Figure 3G–L) [32,33,34,35], closely resembling the pattern of the SkM of tumor hosts (see above). Comparable results were obtained using human differentiated myotubes incubated with C26 or LLC-CM (Table 2), while Dexa treatment exerted a different effect (Table 2).

To evaluate whether the reduced CerK protein expression could derive from the increased degradation promoted by Dexa, prior to be exposed to the GC, C2C12 myotubes were treated with MG132, a proteasome and calpain inhibitor [46,47]. As expected, treatment with the proteasome inhibitor significantly prevented the increase of atrogin-1/MAFbx elicited by Dexa in C2C12 myotubes (Figure 3M,O). As for CerK, MG132 alone was able to increase its expression of approximately 30%, whereas, in the presence of dexa, it enhanced GC-induced CerK down-regulation (Figure 3N,O).

### 3.3. CerK Silencing Induces Atrogin-1/MAFbx Expression in Both In Vivo and In Vitro Models

**Figure 4:**
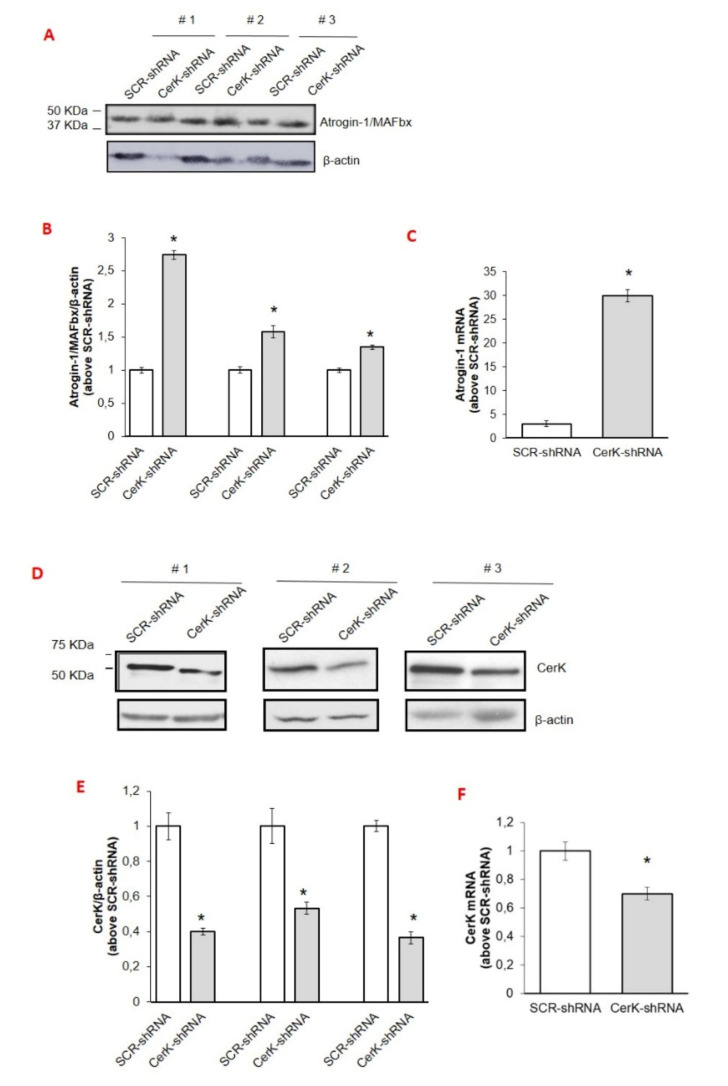
Effects of CerK silencing on atrogin-1/MAFbx and CerK protein and mRNA expression in tibialis muscle tissues (**A**–**F**). Cell lysates for atrogin-1/MAFbx (**A**) and CerK (**D**) and protein expression and total RNA for atrogin-1/MAFbx mRNA expression (**C**) or CerK (**F**) were obtained from tibialis muscle tissues of healthy mice electroporated with scrambled shRNA (SCR-shRNA) or shRNA specific to CerK (CerK-shRNA) as described in Methods. Healthy mice were randomized into groups, SCR-shRNA (*n* = 3) and CerK-shRNA (*n* = 3). Tissue lysate proteins (40 µg), obtained from tibialis muscle tissues of healthy mice transfected with SCR-shRNA or CerK-shRNA, were subjected to SDS-PAGE and immunoblotted as reported in Methods. A blot representative of at least three independent experiments and densitometric analysis is shown (**B**,**E**). Normalized data (means ± SEM) to the β-actin bands, are reported in the graph (Student’s *t* test, * *p* < 0.05 vs. SCR-shRNA). (**C**,**F**): Total RNA was prepared and reverse transcripted and Real-time PCR performed using primers for atrogin-1/MAFbx (**C**) or CerK (**F**) as reported in Methods. Data are presented as fold change (mean ± SEM) of at least 3 independent experiments (Student’s *t* test, * *p* < 0.05 vs. SCR-shRNA).

To confirm the involvement of CerK down-regulation in increasing atrogin-1/MAFbx levels, the expression of CerK was silenced by RNA interference with a pool of three target-specific 19–25 nt shRNAs (CerK-shRNA) in the tibialis anterior (TA) muscle of both control and C26-bearing mice. Consistently with this hypothesis, CerK-silenced muscles were reduced in size (Table 1) and characterized by increased expression of atrogin-1/MAFbx, increased of about two- (protein) and thirty-folds (mRNA; Figure 4A–C), with respect to muscles exposed to the scramble shRNA. Correctness of the experimental conditions was demonstrated by reduced CerK protein and mRNA levels in silenced muscles (Figure 4D–F).

Altogether, these findings indicate that the reduction of CerK expression, observed in tumor-bearing mice and recapitulated in healthy mice by CerK silencing, is associated with the up-regulation of atrogin-1/MAFbx expression and reduced muscle mass, thus confirming the relevance of CerK expression in maintaining a physiological SkM homeostasis. By contrast, CerK silencing did not exert any effect on the TA of tumor-bearing mice (Table 1), suggesting that the proteolytic drive in this condition already have reached the plateau and could not be further stimulated.

**Figure 5:**
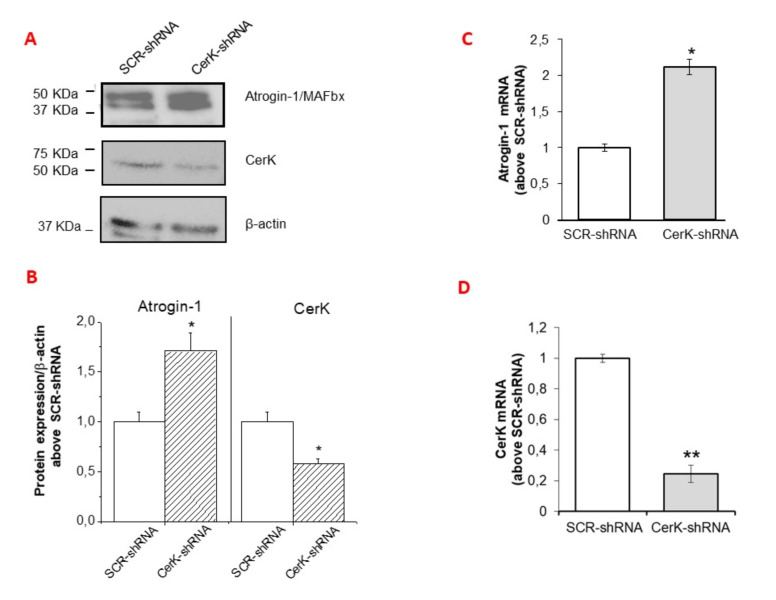
Effect of CerK silencing on atrogin-1/MAFbx and CerK protein and mRNA expression in C2C12 myotubes. (**A**–**D**): cell lysates for CerK and atrogin-1/MAFbx protein expression (**A**) and total RNA for atrogin-1/MAFbx (**C**) or CerK (**D**) mRNA expression were obtained from C2C12 myotubes transfected with scrambled shRNA (SCR-shRNA) or shRNA specific to CerK (CerK-shRNA), as described in Methods. (**A**,**B**): lysates (40 µg) from C2C12 myotubes transfected with SCR-shRNA or CerK-shRNA were subjected to SDS-PAGE and immunoblotted with anti-CerK or anti-atrogin-1/MAFbx antibodies. A blot representative of the densitometric analysis is shown. Normalized data (means ± SEM), to β-actin band, are reported (Student’s *t*-test, * *p* < 0.05 vs. SCR-shRNA). (**C**,**D**): total RNA was purified, reverse transcripted and analyzed as reported in Methods using specific primers for atrogin-1/MAFbx (**C**) or CerK (**D**). Data are presented as fold change (mean ± SEM) of at least 3 independent experiments (Student’s *t* test,* *p* < 0.05, ** *p* < 0.01 vs. SCR-shRNA).

CerK expression knock-down was also obtained by transfecting myotubes with the pool of shRNAs. The CerK silencing efficiency was significant but not complete, achieving a reduction of CerK mRNA and CerK protein content of about 80% and 40% compared to scramble control cells (SCR-shRNA), respectively (Figure 5A,B,D). Although, with a reduced downregulation, similar findings were obtained by transfecting C2C12 cells with siRNA specific for CerK [42] (data not shown). Confirming the initial hypothesis, a significant increase of atrogin-1/MAFbx expression was observed in CerK-silenced cells at both mRNA and protein levels (Figure 5A–C).

### 3.4. Involvement of the Cer/C1P/SphK1 Axis to Dexa-Induced SKM Atrophy in C2C12 Myotubes

**Figure 6:**
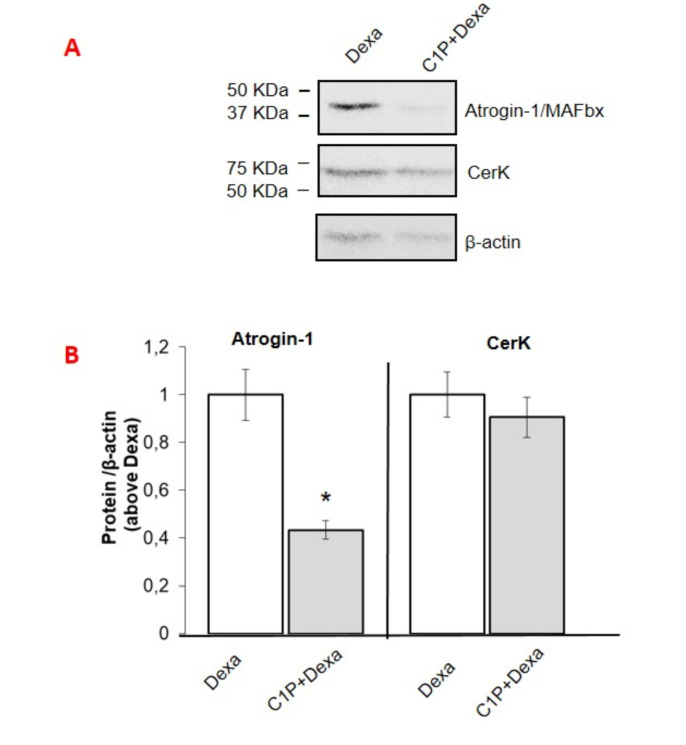

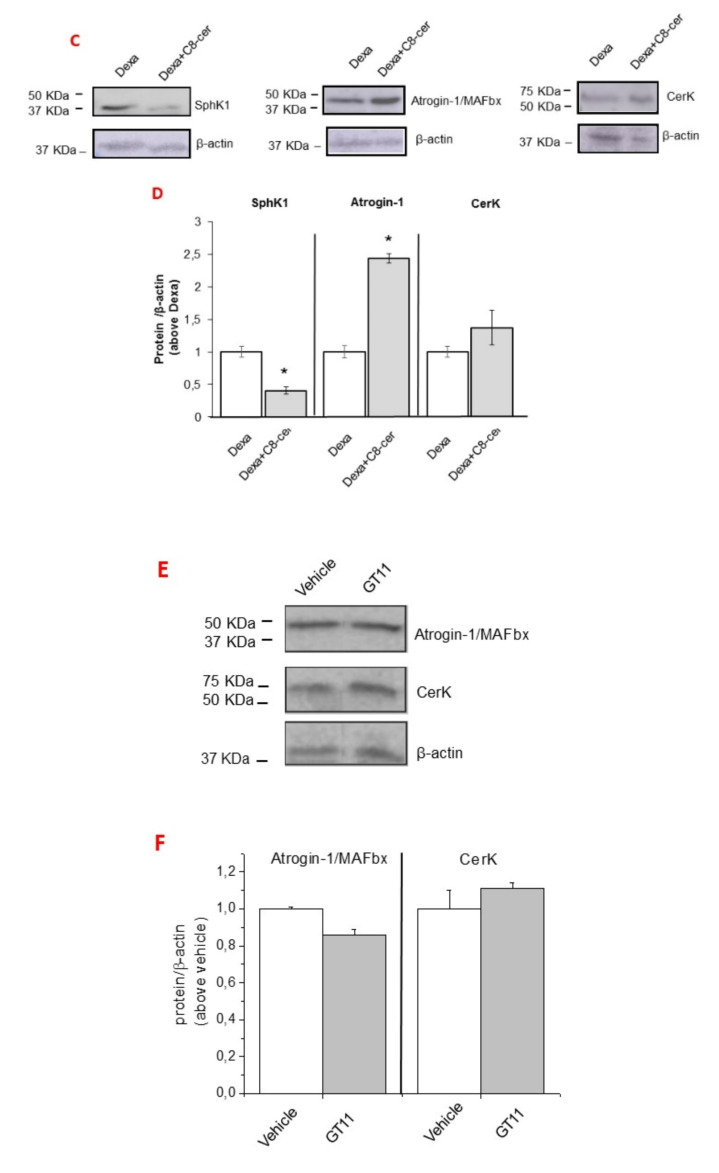
Effect of ceramide 1-phosphate, C8-ceramide and dihydroceramide desaturase inhibitor GT11 on atrogin-1/MAFbx and CerK protein and mRNA expression in C2C12 myotubes. (**A**–**D**): atrogin-1/MAFbx, CerK and SphK1 (**C**) protein expression levels in dexamethasone (Dexa)-treated C2C12 myotubes. C2C12 myotubes were treated with ceramide 1-phosphate (C1P, 2 μM) and C8-ceramide (C8-cer, 10 μM) prior 100 ìM dexa for 48h. Lysates (40 µg of proteins) were subjected to SDS-PAGE, immunoblotted with anti-atrogin-1/MAFbx or anti-CerK antibodies, while cell membrane fractions were used to detect SphK1 protein expression levels. (**E**,**F**): C2C12 myotubes were treated with vehicle (vehicle, DMSO less than 0.05%) or the dihydroceramide desaturase inhibitor (GT11, 10 μM) for 24h. Cells lysates (40 µg) were separated by SDS-PAGE and immunoblotted with anti-atrogin-1/MAFbx or anti-CerK antibodies (**E**). A blot representative of at least three independent experiments and densitometric analysis of atrogin-1/MAFbx, CerK and SphK1 in myotubes treated as above is shown (**D**,**F**). Data (mean ± SEM), normalized to the β-actin band, are reported in the graph relative to dexa (**D**) (Student’s *t* test, * *p* < 0.05 vs. dexa) and to vehicle (**F**).

In order to provide evidence that Dexa effects in myotube cultures require CerK down-regulation, C2C12 myotubes were exposed to the GC in the presence of C1P. As shown in Figure 6A,B, C1P treatment significantly reduced the increase of atrogin-1/MAFbx expression elicited by Dexa, without any change in CerK content, suggesting that the signaling triggered by C1P counteracts the Dexa-induced ubiquitin ligase overexpression. It has been reported by our group that the active phosphorylated form of SphK1 isoform, responsible for S1P synthesis, is reduced in Dexa-treated cells [17]. Moreover, Nishino and collaborators demonstrated that the Cer/C1P axis controls the translocation of the active phospho-SphK1 to membrane in SphK1-overexpressing A549 cells [48].

Therefore, to further investigate the molecular mechanisms of GC action, C2C12 myotubes were treated with dexa and incubated with C8-ceramide (C8-cer), a cell-permeable biologically active Cer analogue, which mimics CerK down-regulation. Exposure to C8-cer significantly reduced the membrane-associated SphK1 (approximately 60%) and increased atrogin-1/MAFbx expression, without modifying CerK content (Figure 6C,D), showing that Cer accumulation mimics dexa effects on the translocation/activation of SphK1.

Finally, to exclude that accumulation of dihydroceramide, the metabolite precursor of Cer, could account for Dexa effects, C2C12 myotubes were incubated with GT11, a specific inhibitor of the dihydroceramide desaturase. The data shown in Figure 6E,F rule out this possibility, since both CerK and atrogin-1/MAFbx expression levels in myotubes exposed to GT11 were comparable to vehicle.

## 4. Discussion

The bioactive SLs and their metabolizing enzymes represent interesting biomolecules involved in many crucial functions in different biological systems [22]. Among them, the SphK/S1P/S1PR axis has received attention for its biological and therapeutic potential [21,49,50,51] in cancer and inflammation. Our group, and others, contributed to clarify the role of the bioactive S1P in SkM cell biology, showing its role as pro-survival and myogenic factor [13,51,52,53,54]. The capability of S1P to also modulate SkM phenotype has been also documented in vivo and ex-vivo/in vitro by our group and others [17,26,55]. Compared with SphK, the information regarding the involvement of CerK and its product C1P in SkM phenotype is limited. CerK was discovered more than 20 years ago in brain synaptic vesicles [56] and, to date, it remains the only kinase known in mammalian cells to convert Cer to C1P. CerK is ubiquitously expressed in various mammalian tissues [24] and its importance only recently started to be appreciated [28,57,58] in cancer as well as in other pathological states, such as those characterized by inflammation. In particular, CerK has been found overexpressed in breast cancer and its level is associated with poor prognosis [57,59]. In addition, the CerK/C1P axis is able to promote lung cancer cell growth and survival and pancreatic cancer cell migration and invasion [60]. The connection between CERK/C1P and cPLA2α activation has been reported and is of great interest with regard to several diseases [23]. Changes in Cer metabolism have been linked with SkM wasting upon short-term unloading [61], aging [62] and in skeletal muscle atrophy induced by TNF-α [26]. A role of Cer has also been reported in other diseases characterized by tissue atrophy, such as heart failure [59,63].

Here, we have extended our knowledge on the involvement of the CerK/C1P in the acquisition and maintenance of mature SkM phenotype, providing evidence of a down-regulation of CerK either in SkM tissue obtained from cachectic mice as well as in different muscle cellular systems exposed to CM of C26/LLC cultures or to dexa.

The observation that, in mice bearing the C26 or LLC tumor the down-regulation of CerK is associated with body weight loss, muscle mass depletion and atrogin-1/MAFbx overexpression, unravels a new potential pathogenetic mechanism accounting for the alterations of muscle homeostasis in cancer cachexia. This is further supported by the up-regulation of atrogin-1/MAFbx expression observed in TA muscles of healthy animals, in which CerK expression was down-regulated by specific shRNA transfection. Therefore, the negative regulation of CerK expression may contribute to the cancer-induced SkM atrophy in vivo, potentially acting on both differentiated myofibers and SkM progenitor cells.

The data obtained in C2C12 cells indicate the relevance of CerK functions both in myoblasts and in terminally differentiated myotubes. In fact, the pharmacological inhibition of CerK promotes myoblast growth arrest, indicating that cell entry in G2/M cell cycle phase requires CerK activity. However, CerK inhibitors did not promote the expression of myogenic markers (Meacci, personal communication), demonstrating that the balance between Cer and C1P level is not sufficient to boost myogenesis. Finally, these observations show that the expression of CerK is required for proper myoblast differentiation and for maintaining the terminal differentiated phenotype in myotubes.

Further studies are needed in order to better clarify the underlying mechanisms leading to impaired CerK expression in vivo/in vitro and to identify potential targets for counteracting CerK loss and, eventually, muscle atrophy.

In acute tissue atrophy, it is known that the expression of the ubiquitin ligase atrogin-1/MAFbx [1,64] is associated with increased protein degradation. Consistently, we found that dexa-induced atrogin-1/MAFbx overexpression parallels the reduction of CerK protein but not mRNA content, suggesting an impaired CerK turnover in GC-treated cells.

Although the regulation of CerK expression and protein stability remain scarcely investigated, few studies reported the involvement of the transcription factor chicken ovalbumin upstream promotor transcription factor I (COUP-TFI). For instance, in human neuroblastoma cells, COUP-TFI is required for all-transretinoic acid (ATRA)-induced inhibition of CerK transcription [42]. We previously reported that in retinoic acid-differentiated neuroblastoma cells, CerK expression is negatively regulated by vitamin D signaling, and COUP-TFI-silencing is able to rescue hormone effect. However, in preliminary experiments, we found that COUP-TFI-silencing does not prevent CerK downregulation in C2C12 myotubes. In retinoic acid-differentiated neuroblastoma cell system, the proteasome inhibitor MG132 promotes the increase of the basal expression of CerK [65]. Similarly, here we reported that MG132 treatment induces a strong increase of CerK protein expression in myotubes, while in the presence of Dexa, CerK levels were lower with respect to control cultures.

These findings support the idea that, while proteasome activity is required to keep low the expression of CerK in normal cells, it does not appear involved in dexa-induced effects on the enzyme, since this latter is not counteracted by MG132. A possible explanation of our findings is the ability of Dexa to control CerK protein stability by involving a different protein degradation system, such as calpain, or to activate other signaling pathways involved in cell toxicity/apoptosis [66]. Moreover, MG132 is able to trigger many signaling events other than merely inhibiting proteasome; for example, it can down-regulate NF-κB activation, [67,68]. The role of NF-κB signaling on CerK expression and other proteolytic systems may be worth to be investigated. In addition, CerK was recently reported to behave as a target of microRNA34a, which is increased in SkM of 2-year-old mice and in the myoblasts of insulin-resistant humans [62]. The miR34a role in the regulation of CerK expression in the experimental conditions of this study is under investigation.

Other interesting findings of this study are the evidence that, in dexa-treated cells, C8-cer treatment further enhances atrogin-1/MAFbx expression and reduces the membrane translocation of the active SphK1. This limits S1P generation, further supporting the role of the Cer/C1P axis in the accomplishment of SkM atrophy. Therefore, since we previously demonstrated that the atrophic phenotype of myotubes treated with dexa is strictly correlated with the impairment of S1P signaling [17], we propose a novel mechanism by which the functional crosstalk between CerK/C1P and SphK1/S1P controls SkM cell atrophy in GC-treated cells. Indeed, the downregulation of CerK not only affects C1P formation, but also S1P synthesis. This event may contribute to further reduce C1P content since additional anabolic pathways of C1P have been reported implicating sphingomyelinase D-mediated transfer of fatty acyl chain to S1P [69].

## 5. Conclusions

**Figure 7:**
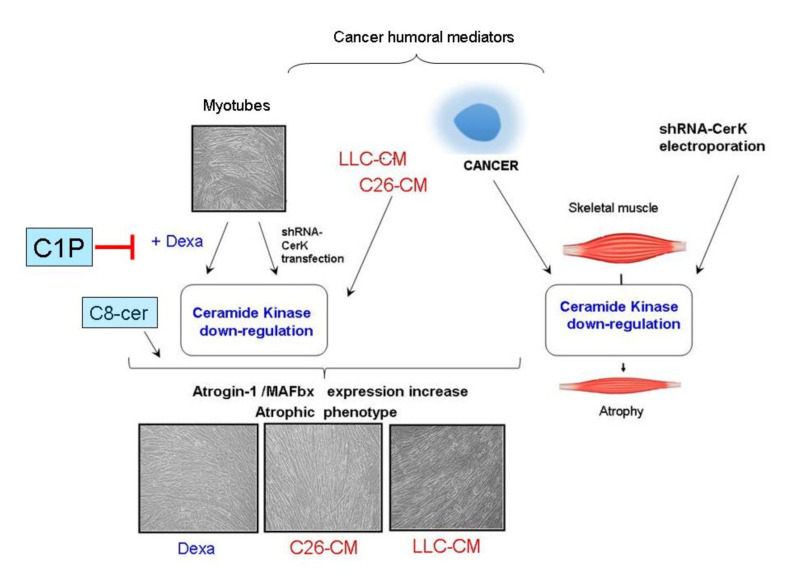
Schematic drawing representing the potential influence of CerK in the context of skeletal muscle atrophy associated with cancer or corticosteroids. Abbreviations: C1P, ceramide 1-phosphate; Dexa, dexamethasone; C8-cer, C8-ceramide; CerK-shRNA, short hairpin RNAs targeting CerK mRNA; C26-CM, conditioned media from C26 carcinoma cells; LLC-CM, conditioned media from Lewis lung carcinoma cells.

In conclusion, we demonstrate that CerK is expressed in myoblasts and in murine and human skeletal muscle differentiated cells, thus indicating a specific functional role of the enzyme for the biology of committed cells as well as mature SkM cells (Figure 7).

The latter conclusion is supported by the main observations, and in vivo as well as in vitro model: (a) CerK protein is markedly down-regulated in the SkM of cachectic C26 and LLC hosts and in myotubes treated with conditioned medium from cancer cells or with Dexa; (b) a downregulation of CerK at the mRNA level has also been observed in SkM tissue samples as well as in human primary SkM differentiated cells and murine C2C12 myotubes treated with conditioned medium from cancer cells, but not with the corticosteroid; (c) CerK gene silencing results in the upregulation of atrogin-1/MAFbx, which is generally associated with enhanced protein breakdown rates underlining the role of the CerK/C1P axis to control the ubiquitin ligase expression; (d) in Dexa-treated myotubes the exposure to C8-cer contributes to the decrease in SphK1 active form, while C1P treatment rescues the increase in atrogin-1/MAFbx expression elicited by the GC. These latter findings point out the existence of a crosstalk between the CerK/C1P and SphK/S1P axis and a new potential therapeutic target for SkM atrophy associated with corticosteroid treatment. Protective effects of C1P have been reported in various contexts. For example, knockout of CerK aggravates the pathology in mice with experimental colitis [70], C1P exerts protective effects by determining the up-regulation of prostanoids, and down-regulates, in different cell systems, the secretion of pro-inflammatory cytokine TNF-α [26]. This latter observation also supports those modulations of CerK activity significantly impinge on inflammation, further contributing to SkM wasting.

## Figures and Tables

**Table 1:** Characterization of C26-bearing mice vs. control animals and CerK silencing on tibialis anterior weight.

Parameter	C	C26 Hosts
Initial body weight (g)	19.16 ± 0.50	18.86 ± 0.97
Final body weight (g)	21.83 ± 0.8	17.17 ± 1.2 ***
Gastrocnemius (mg/100g i.b.w.) left	556 ± 18	415 ± 47 **
Gastrocnemius right (mg/100g i.b.w.)	563 ± 30	431 ± 51 ***
Liver (mg/100g i.b.w.)	5932 ± 418	5378 ± 472 *
Spleen (mg/100g i.b.w.)	420 ± 32	1220 ± 296 ***
Heart (mg/100g i.b.w.)	546 ± 32	517 ± 57
Tumor mass (mg)	-	351 ± 56
Tibialis anterior (mg/100g i.b.w.)		
SCR-shRNA	228 ± 29	134 ± 32 ***
CerK-shRNA	170 ± 16 ***	119 ± 28

Mice of about 8 weeks were randomized into two groups, namely C (controls, *n* = 8) and C26 hosts (*n* = 10); i.b.w.: initial body weight. Further details can be found in Materials and Methods section. Data are means ± SD. Significance of the difference: * *p* < 0.05, ** *p* < 0.01, *** *p* < 0.001 vs. C.

**Table 2:** Effect of Desamethasone or of conditioned medium derived from LLC or C26 cells on CerK and atrogin-1/MAFbx mRNA expression in human primary skeletal muscle differentiated cells.

CerK mRNA (Above Vehicle ± S.E.M.)		Atrogin-1 mRNA (Above Vehicle ± S.E.M.)	
Vehicle	1 ± 0.04	Vehicle	1 ± 0.2
Dexa	1.2 ± 0.12	Dexa	6 ± 0.8 **
DMEM 1:4	1 ± 0.1	DMEM 1:4	1 ± 0.1
CM-LLC 1:4	0.55 ± 0.16 *	CM-LLC 1:4	5.2 ± 0.51 **

Total RNA for CerK and atrogin-1/MAFbx mRNA were obtained from untreated (vehicle, DMSO less than 0.05%) or dexamethasone-treated human primary skeletal muscle differentiated cells (Dexa) or cells cultured with DMEM (control group) or with conditioned medium obtained from LLC (CM-LLC) as described in Methods and above. Data are presented as fold change (mean ± SEM) of at least three independent experiments (Student’s *t* test, * *p* < 0.05 vs. DMEM; ** *p* < 0.01 vs. vehicle or DMEM).

## Supplementary Materials

The following are available online at https://www.mdpi.com/article/10.3390/cancers13133285/s1, Figure S1: Uncropped Figure 1A,D (controls), Figure S2: Uncropped Figure 1A,D (C26), Figure S3: Full unedited gel for Figure 1G, Figure S4: Full unedited gel for Figure 2A (left) and Figure 2C (right), Figure S5: Full unedited gel for Figure 3A (left), Figure 3D (right) and Figure 3A–D (down), Figure S6: Uncropped Figure 3G, Figure S7: Full unedited gel for Figure 3M (left) and Figure 3N (right), Figure S8: Full unedited gel for Figure 4A, Figure S9: Full unedited gel for Figure 4D, Figure S10: Full unedited gel for Figure 5A, Figure S11: Full unedited gel for Figure 6A, Figure S11: Full unedited gel for Figure 6C, Figure S12: Full unedited gel for Figure 6C (continued), Figure S13: Full unedited gel for Figure 6E.
