# Supplementary material for: Control of Skeletal Muscle Atrophy Associated to Cancer or Corticosteroids by Ceramide Kinase
Source: Cancers (Basel). 2021 Jun 30;13(13):3285. doi: 10.3390/cancers13133285 (PMC8269416; doi:10.3390/cancers13133285)
Supplement: Supplementary file 1 [file cancers-13-03285-s001.zip › cancers-1244370-supplementary.pdf]

cancers

MDPI

Supplementary Material: Control of Skeletal Muscle Atrophy Associated to Cancer or Corticosteroids by Ceramide Kinase

Federica Pierucci, Alessia Frati, Chiara Battistini, Fabio Penna, Paola Costelli and Elisabetta Meacci

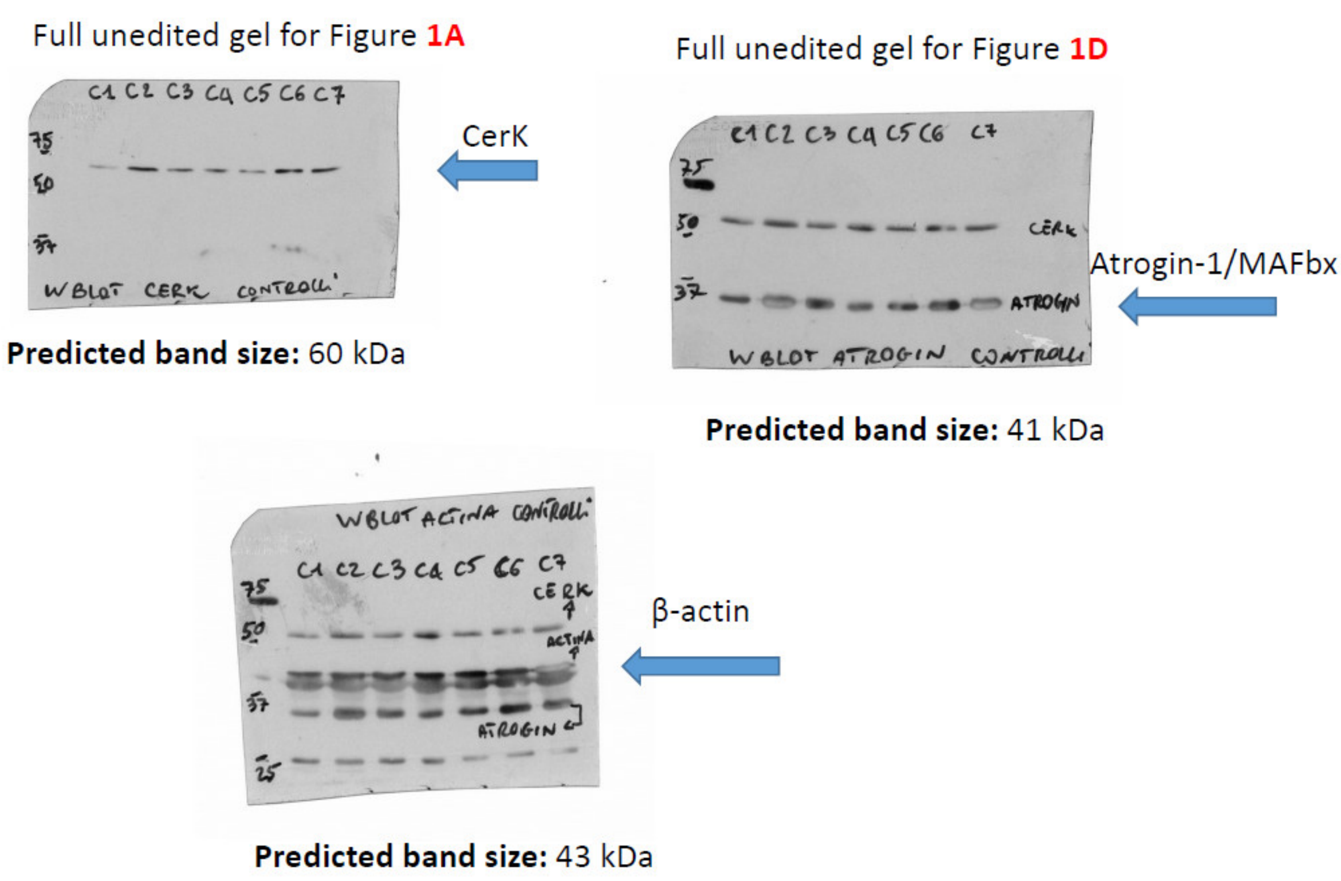

Figure S1. Uncropped Figure 1A,D (controls).

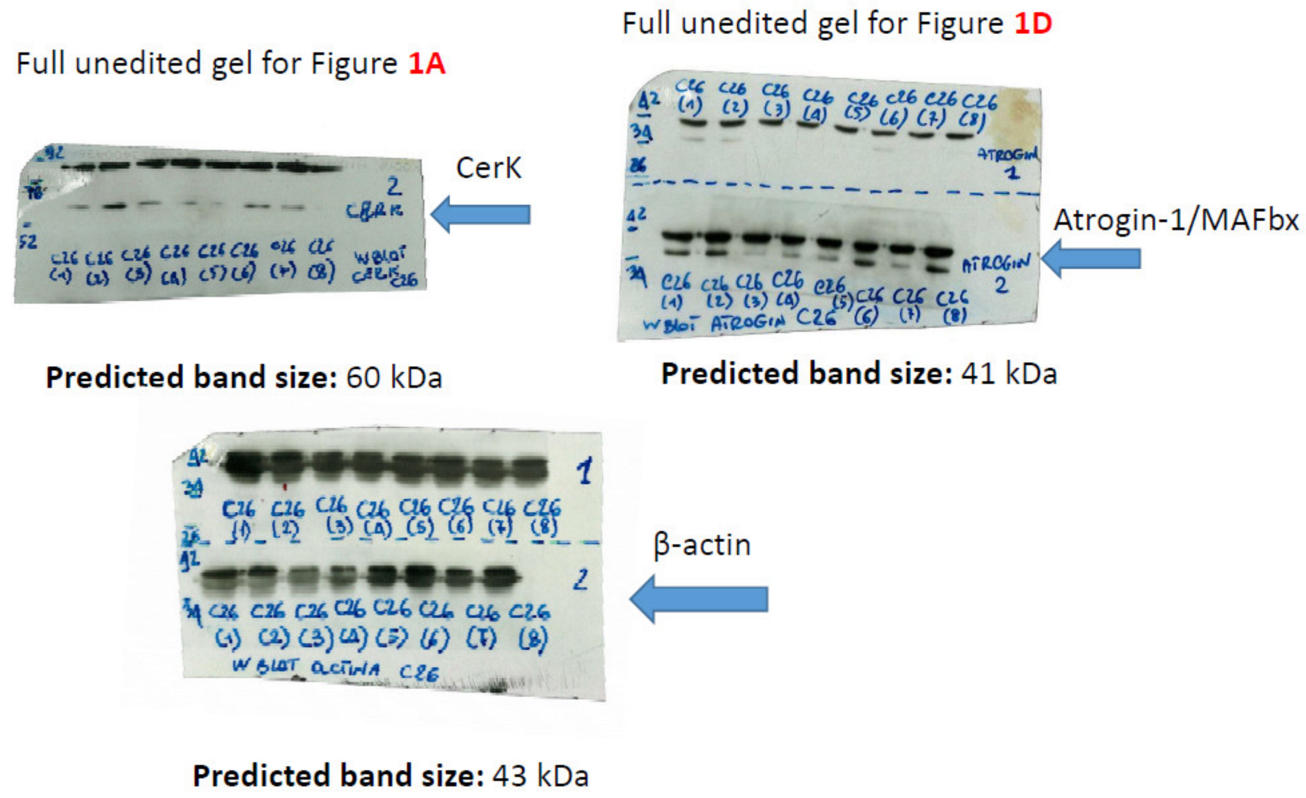

Figure S2. Uncropped Figure 1A,D (C26).

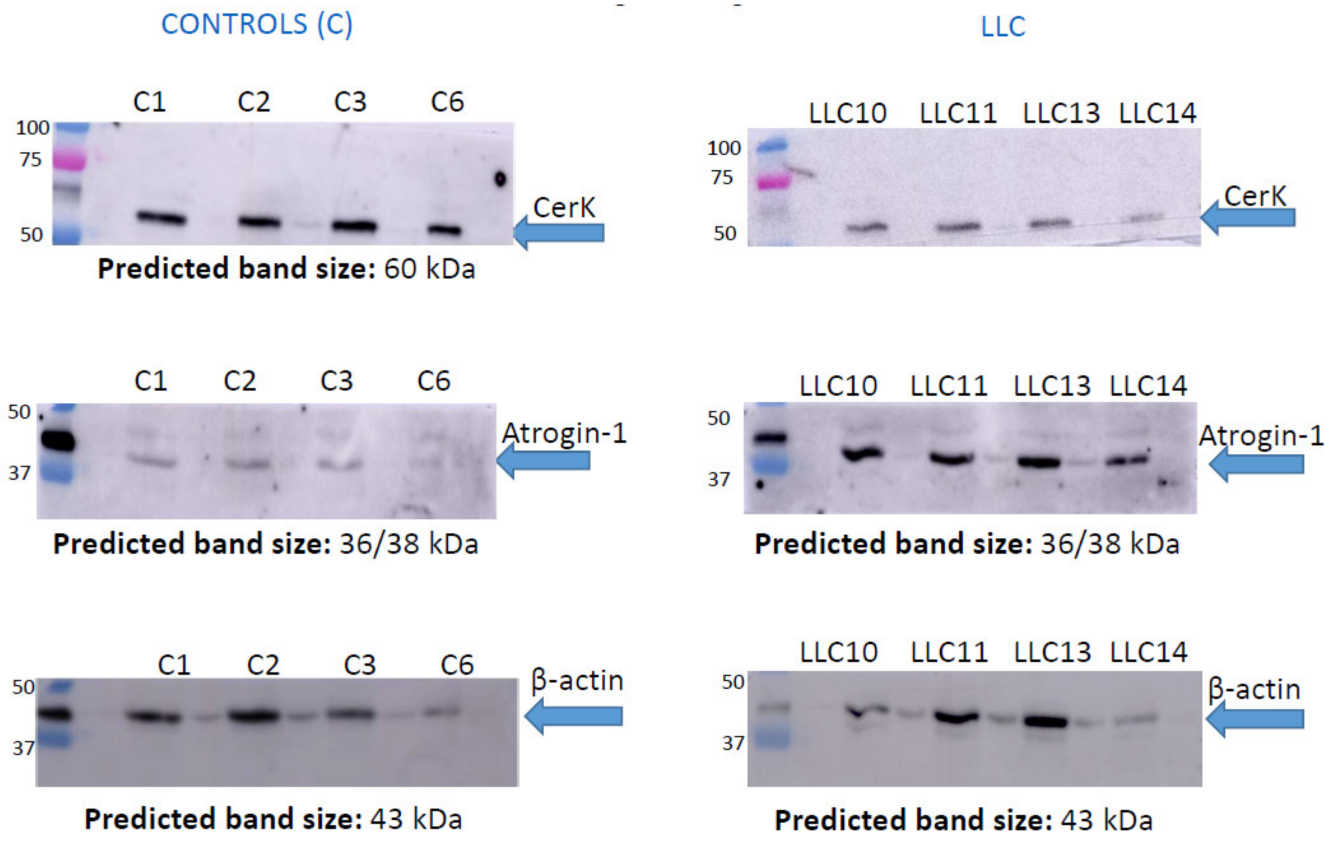

Figure S3. Full unedited gel for Figure 1G.

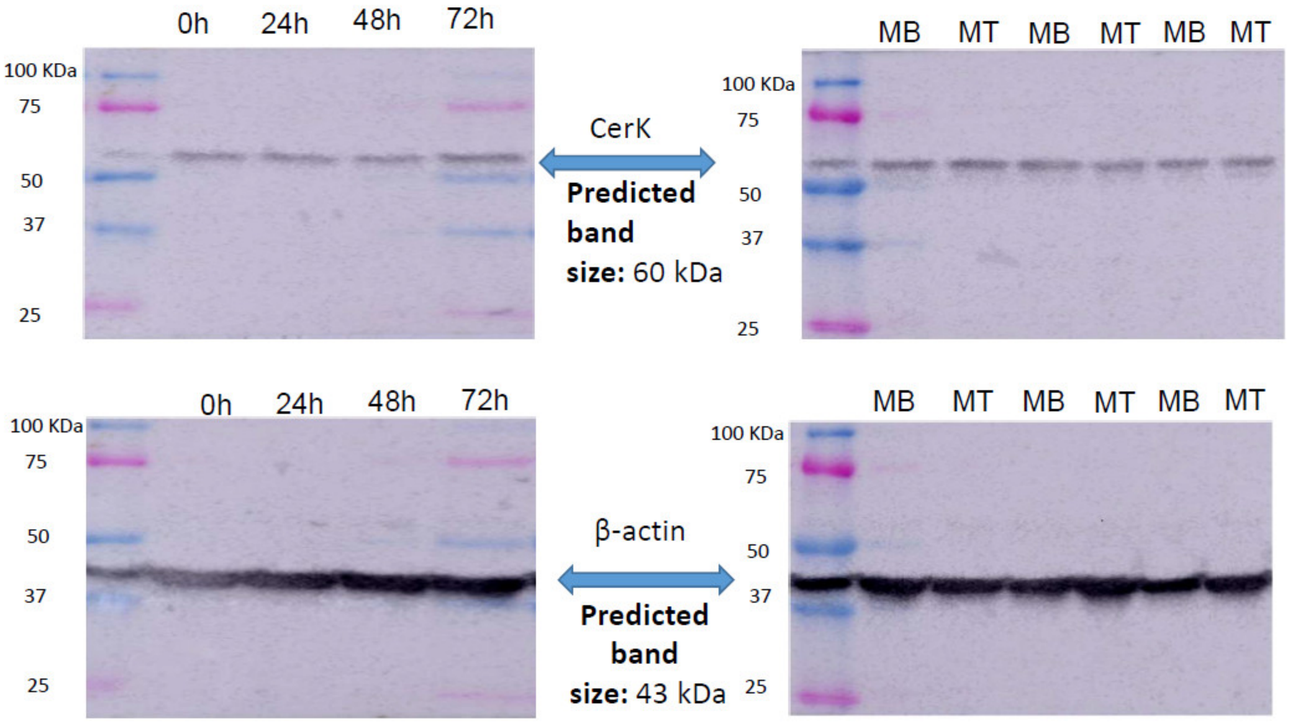

Figure S4. Full unedited gel for Figure 2A (left) and Figure 2C (right).

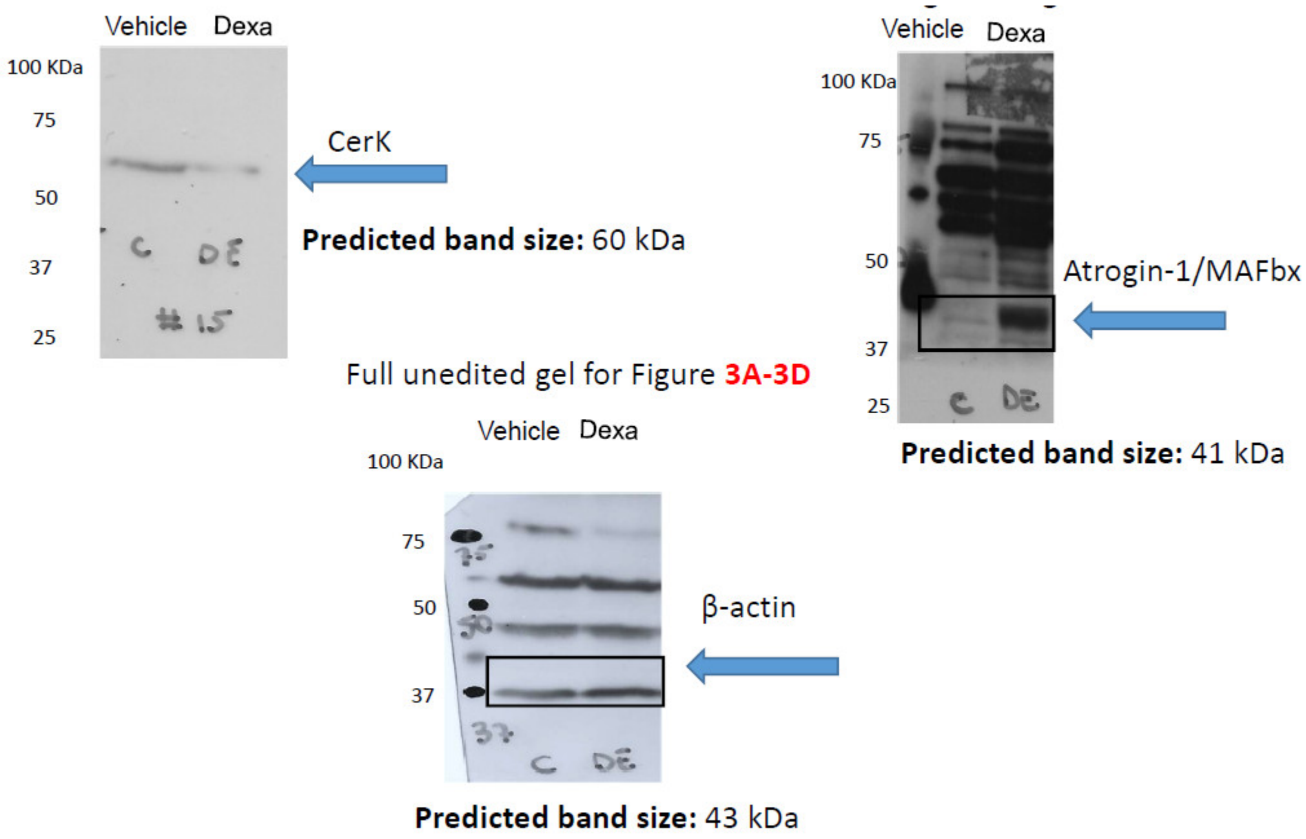

Figure S5. Full unedited gel for Figure 3A (left), Figure 3D (right) and Figure 3A-D (down).

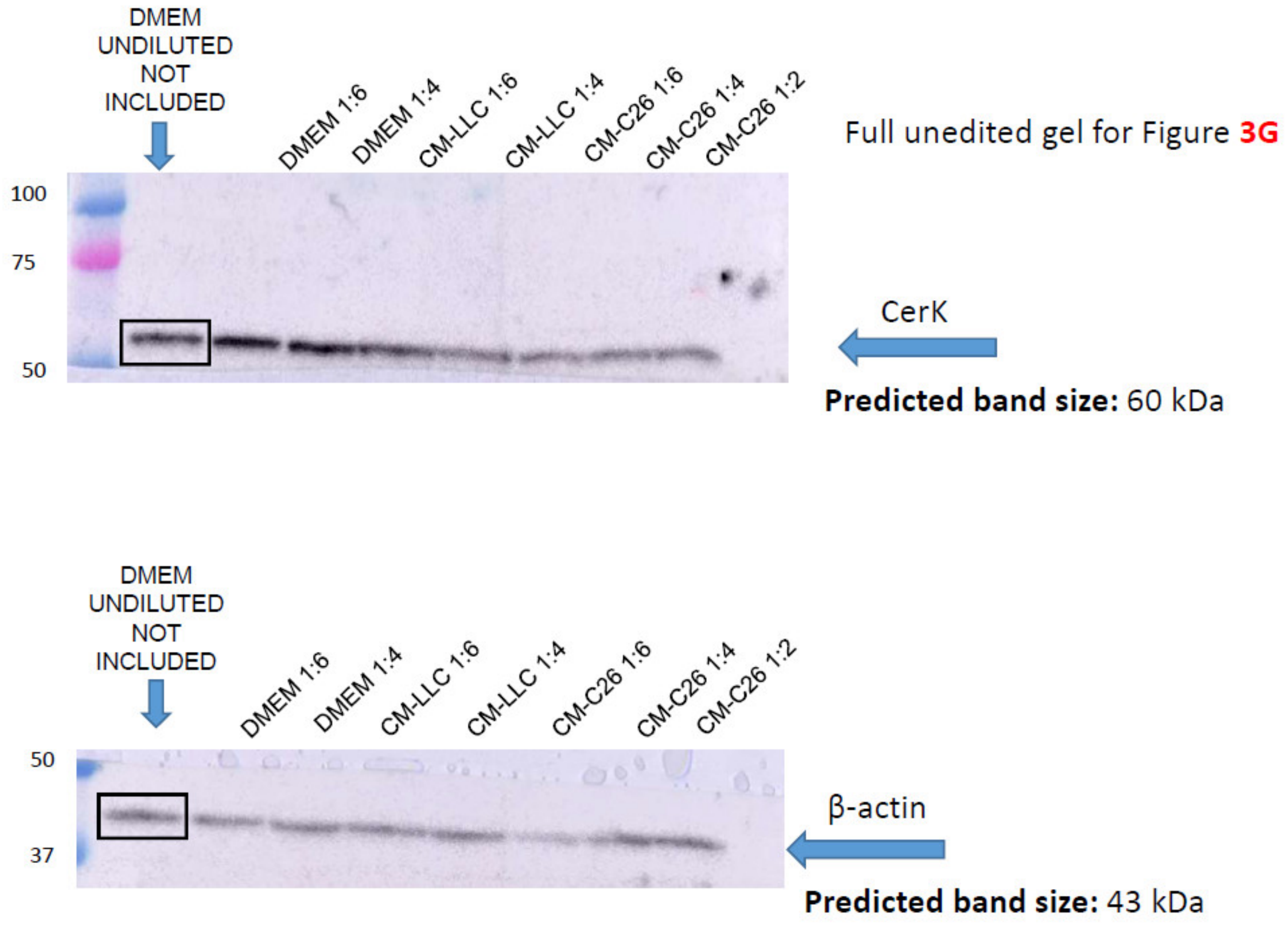

Figure S6. Uncropped Figure 3G.

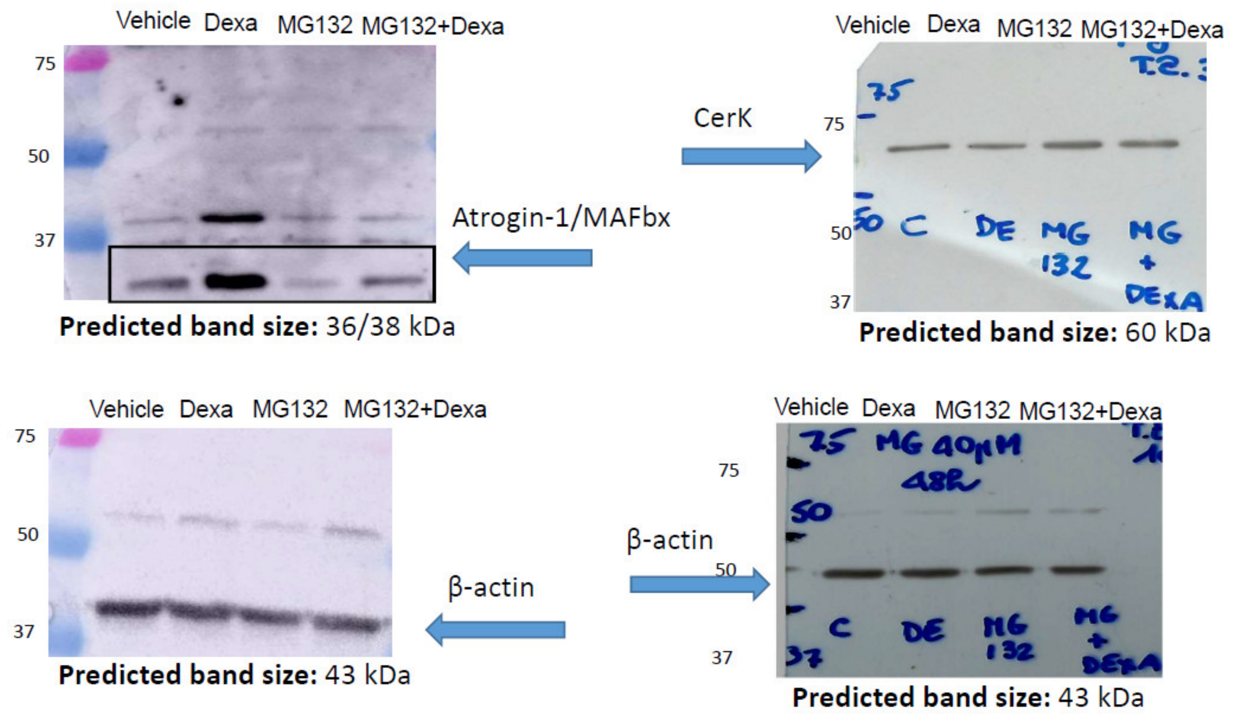

Figure S7. Full unedited gel for Figure 3M (left) and Figure 3N (right).

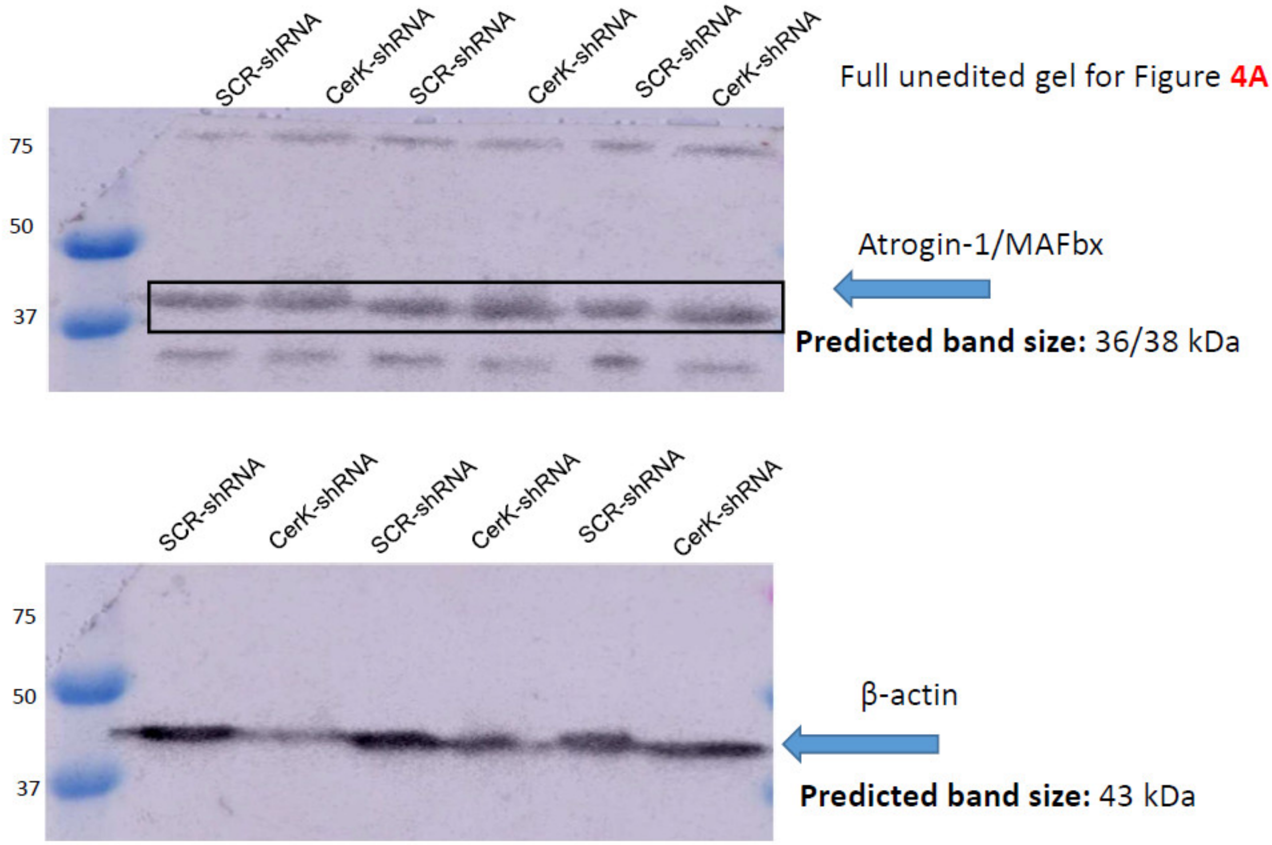

Figure S8. Full unedited gel for Figure 4A.

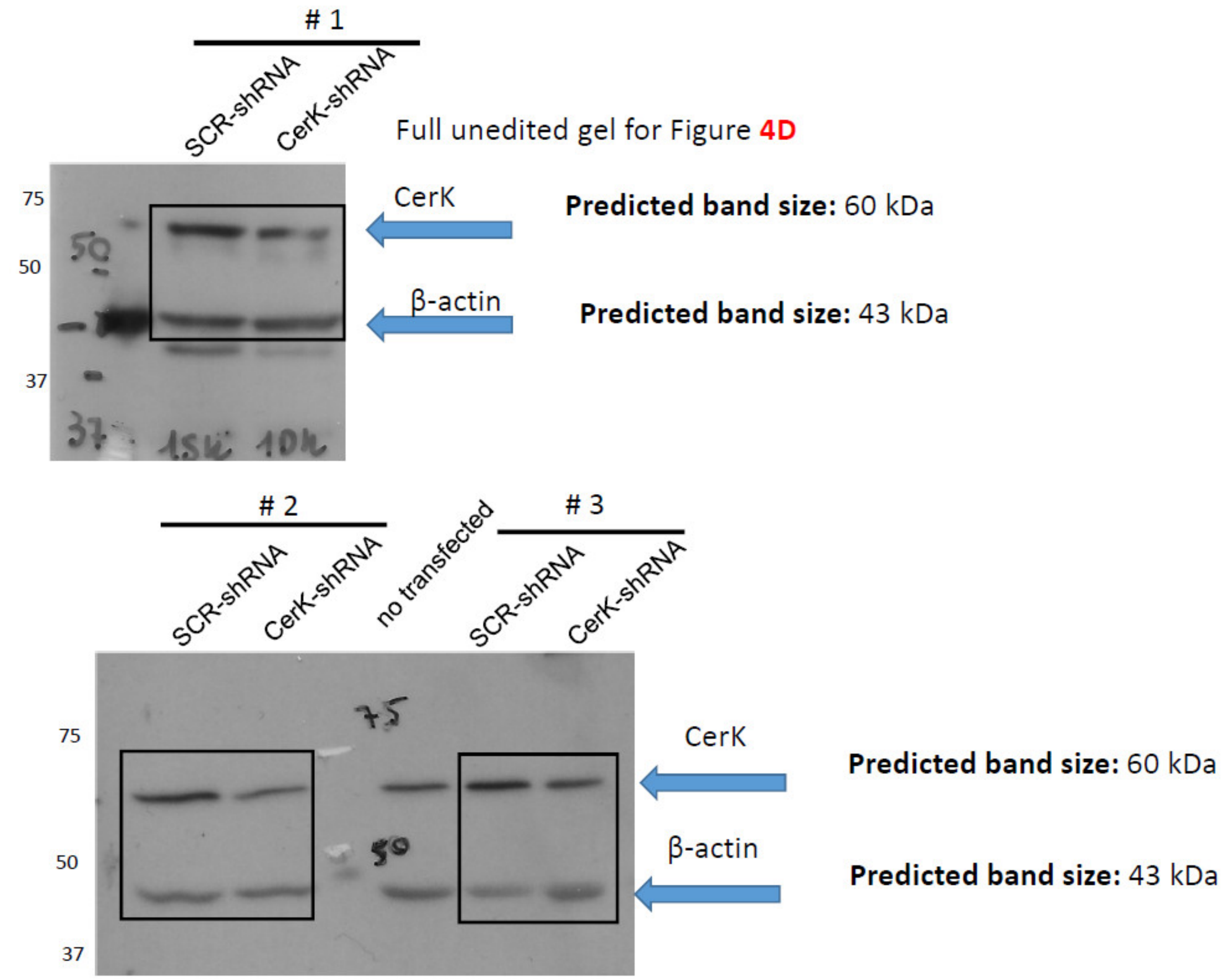

Figure S9. Full unedited gel for Figure 4D.

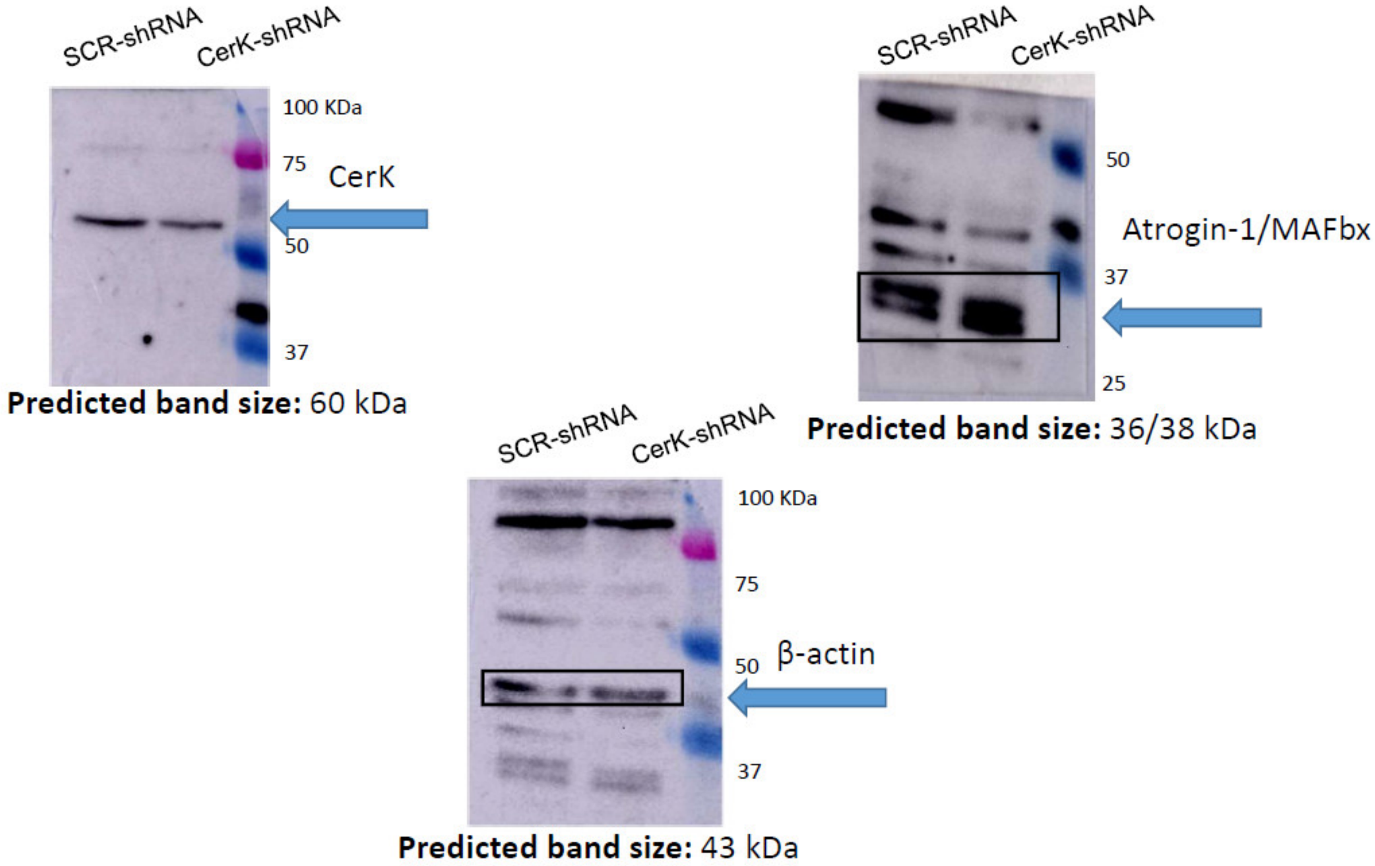

Figure S10. Full unedited gel for Figure 5A.

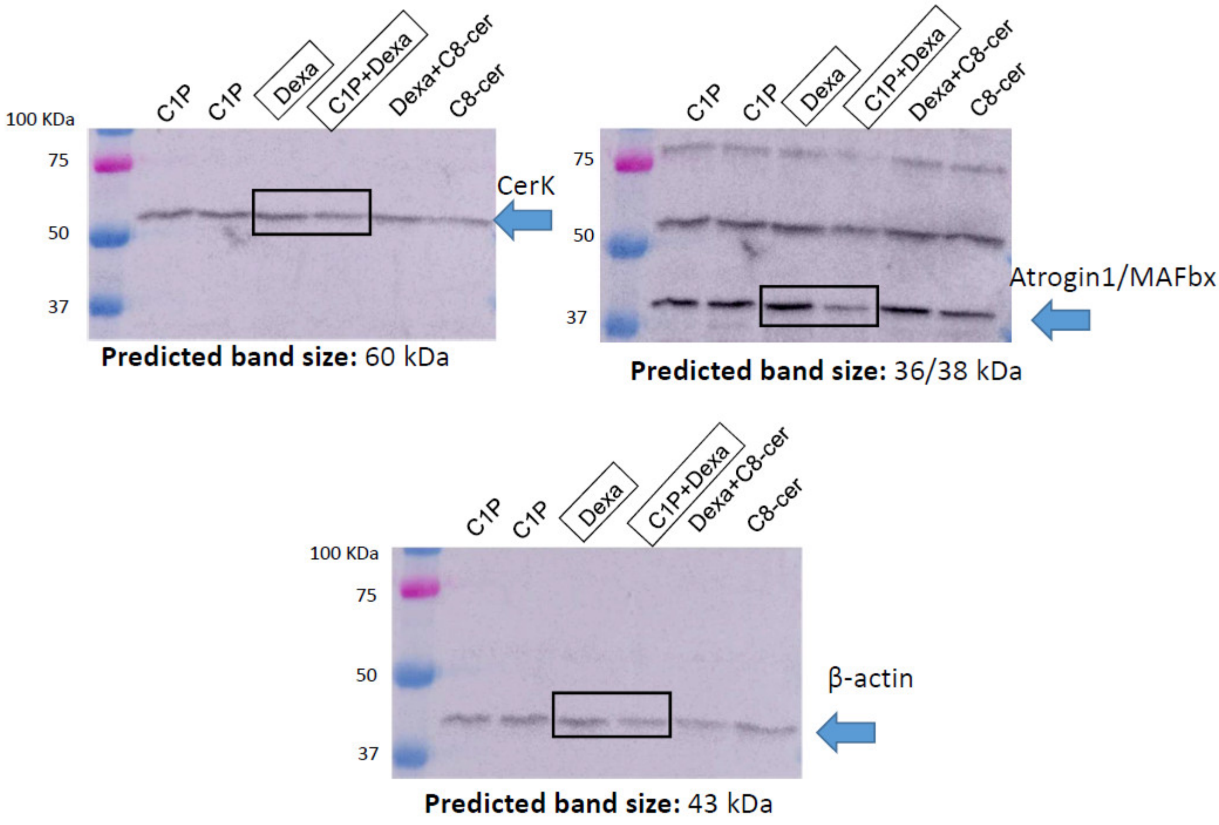

Figure S11. Full unedited gel for Figure 6A.

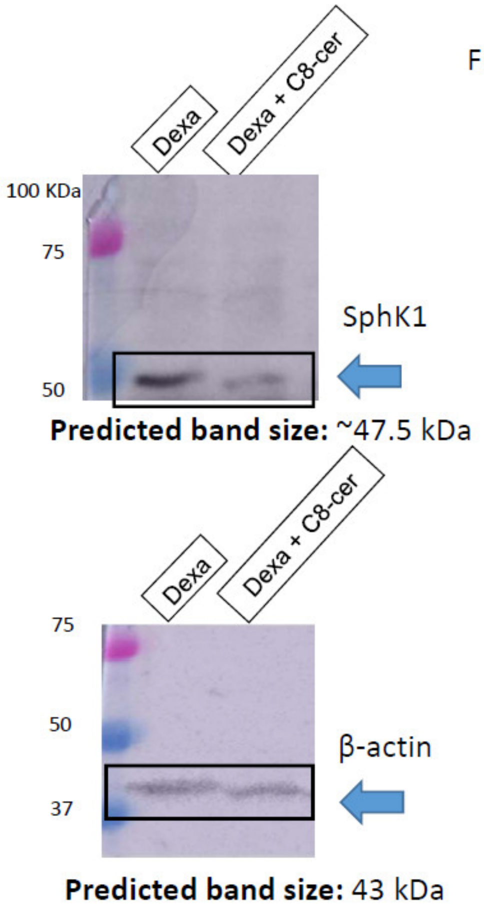

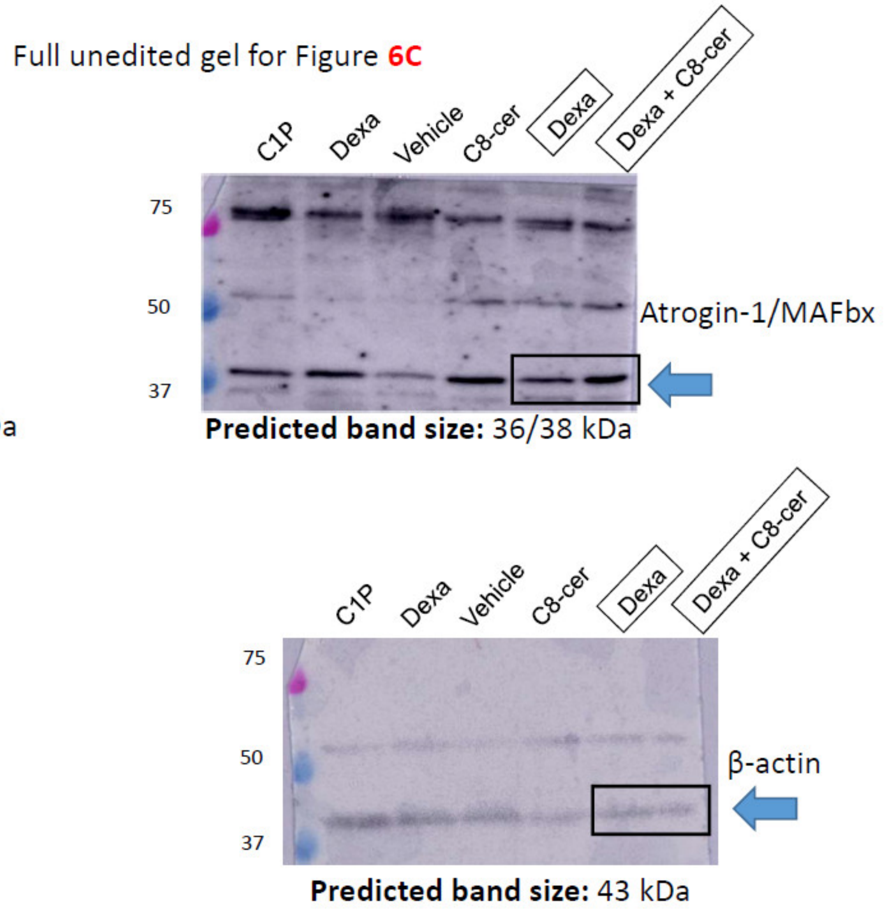

Figure S11. Full unedited gel for Figure 6C.

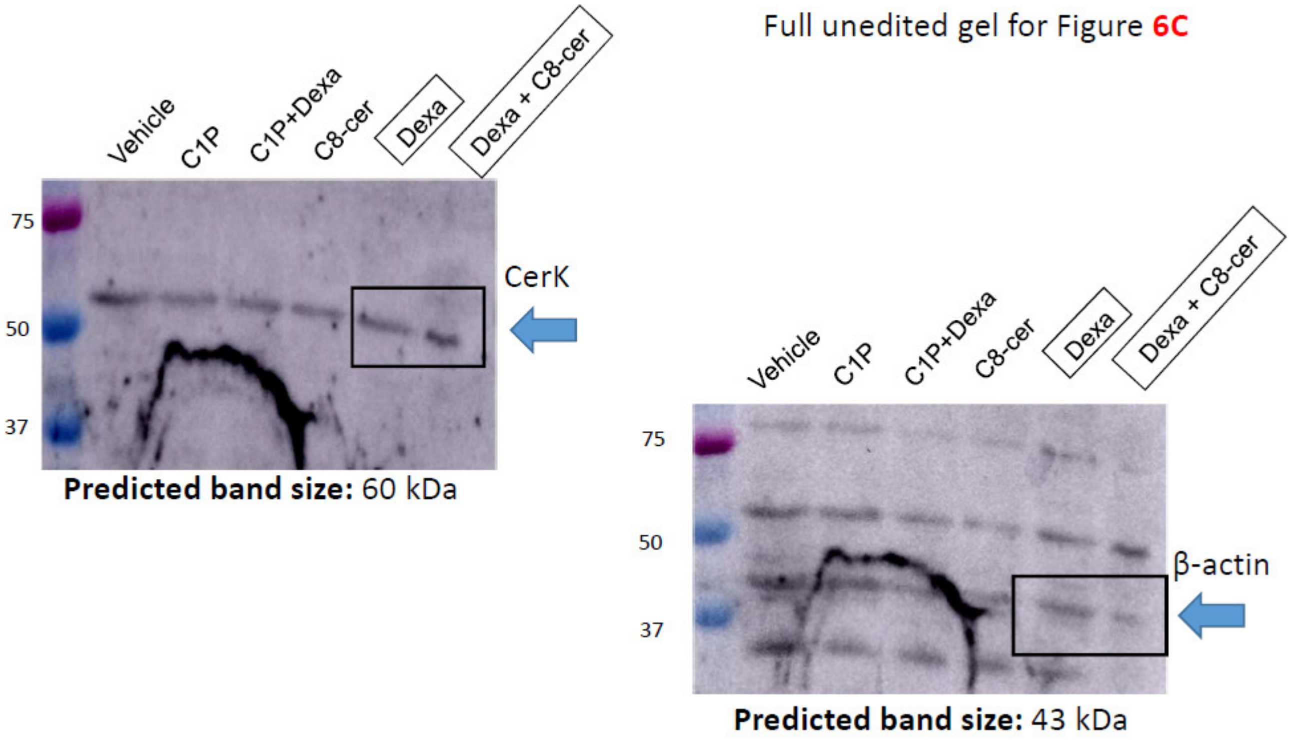

Figure S12. Full unedited gel for Figure 6C (continued).

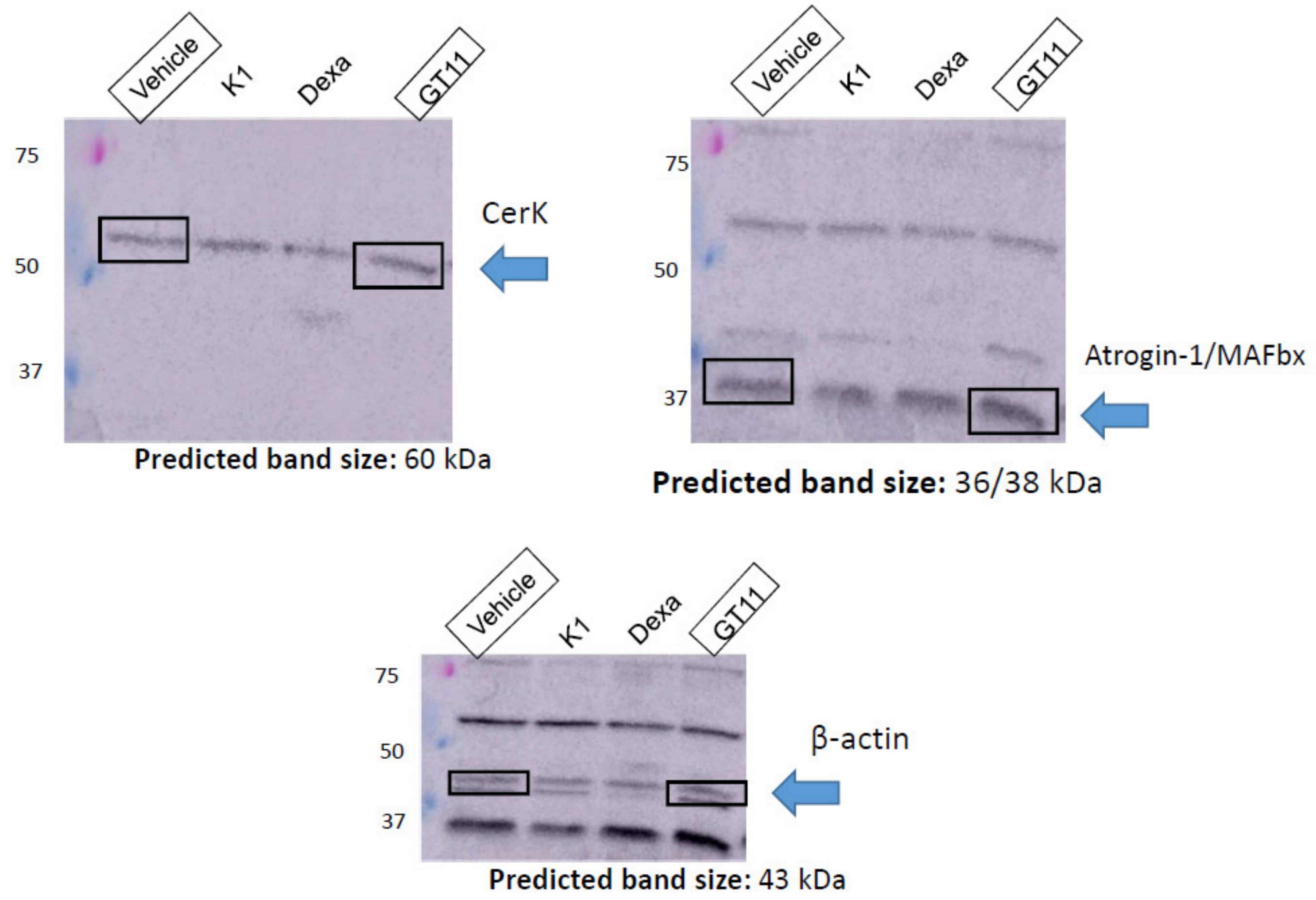

Figure S13. Full unedited gel for Figure 6E.
